# Reference values of gait characteristics in community-dwelling older persons with different physical functional levels

**DOI:** 10.1186/s12877-022-03373-0

**Published:** 2022-08-29

**Authors:** Ulrike Dapp, Dominic Vinyard, Stefan Golgert, Sebastian Krumpoch, Ellen Freiberger

**Affiliations:** 1 Scientific Department at the University of Hamburg, Albertinen-Haus Geriatrics Center, Sellhopsweg 18-22, 22459 Hamburg, Germany; 2 Institute for Biomedicine of Aging (IBA), Friedrich-Alexander-University Erlangen-Nürnberg (FAU), Kobergerstr. 60, 90408 Nürnberg, Germany

**Keywords:** Gait characteristics; community-dwelling, Aged, Physical function, Reference values, Frailty, Prevention, Population-based, Spatiotemporal gait parameters

## Abstract

**Background:**

Mobility is one major component of healthy ageing of older persons. It includes gait speed, nowadays valued as the sixth vital sign of ageing. Quantitative gait analysis can support clinical diagnostics, monitor progression of diseases and provide information about the efficacy of interventions. Fast gait speed is an additional marker in the area of functional ability. Our aim was to contribute reference values of gait parameters of older persons based on their functional ability.

**Methods:**

We visualised and combined three different established frameworks that assess gait characteristics into a new framework based approach that comprises eight gait parameters: gait speed, stride length, walk ratio, single and double support time, step width, step width CV (coefficient of variance), stride length CV. Gait parameters were stratified by two instruments that indicate levels of functional ability: First, the LUCAS Functional Ability Index (FAI), a self-administered screening tool easy to apply to a public-health orientated approach and second the Short Physical Performance Battery (SPPB), an established performance test widely used in comprehensive geriatric assessments (CGA). Gait parameters of older community-dwelling persons were measured with an objective Gait system (GAITRite) across differing functional ability ranging from robust to transient (postrobust and prefrail) to frail physical status.

**Results:**

Of 642 community-dwelling participants (age 78.5 ± 4.8; *n* = 233 male, *n* = 409 female) categorisations by SPPB were 27.1% for robust (11–12 points), 44.2% for transient (8–10 points), 28.7% for frail (0–7 points), and 16.2, 50.3, 33.5% for robust, transient, frail by LUCAS FAI. Overall, our results showed that distinction by functional level only uncovers a wide spectrum of functional decline for all investigated gait parameters. Stratification by functional ability (biological age) revealed a greater range of differentiation than chronological age.

**Conclusions:**

Gait parameters, carefully selected by literature, showed clinically meaningful differences between the functional featuring a gradient declining from robust over transient to frail in most gait parameters. We found discriminative power of stratifications by SPPB to be the highest, closely followed by LUCAS FAI, age groups and dichotomous age making the application of the LUCAS FAI more cost and time effective than conducting SPPB.

**Supplementary Information:**

The online version contains supplementary material available at 10.1186/s12877-022-03373-0.

## Background

Due to demographic changes new concepts and awareness regarding healthy ageing have come into focus of ageing research [1, 2] and the WHO has declared the years 2021–2030 as “The United Nations Decade of Healthy Ageing” (https://www.who.int/initiatives/decade-of-healthy-ageing). Healthy Ageing is defined by the WHO “as the process of developing and maintaining the functional ability that enables well-being in older age”. Functional ability “comprises the health-related attributes that enable people to be and to do what they have reason to value” [1]. Healthy Ageing is based around the concept of functional ability integrating intrinsic capacity (the composite of physical and mental capacities of a person), acknowledging the interaction between physical function over the life span and environmental aspects [1–4]. The concept of intrinsic capacity covers five domains: locomotor, cognitive, psychological, sensory, and vitality. One essential component of locomotion is gait in older persons.

Gait speed is already valued as the sixth vital sign of ageing [5] due to its high predictive value for mortality [6, 7] or dementia [8] and other negative health outcomes [2, 9]. Gait can often be negatively affected by various intrinsic capacities domains, e.g. by impaired vision or cognition, depression or fear of falling [10–12]. Therefore, quantitative gait analysis can support clinical diagnostics, monitor progression of diseases and provide information about the efficacy of interventions [13].

Measures of gait are not limited to gait speed but also extend to other spatio-temporal gait characteristics. Variables such as step-length, step-width, double or single support time or the variance of these parameters, cadence or walk ratio provide further as well as in-depths information on gait quality and the functional level of the tested older person [13–15]. Furthermore, gait is often paced at normal (preferred) gait speed although this does not reflect the affordances of higher gait speed needed in certain daily activities e.g. in traffic situation for crossing a street in the green phase in time or for hurrying to answer the phone or door bell [16]. Therefore, the capacity of older persons to change from normal (preferred) gait speed to fast gait speed is an additional marker of functional ability.

**Fig. 1:**
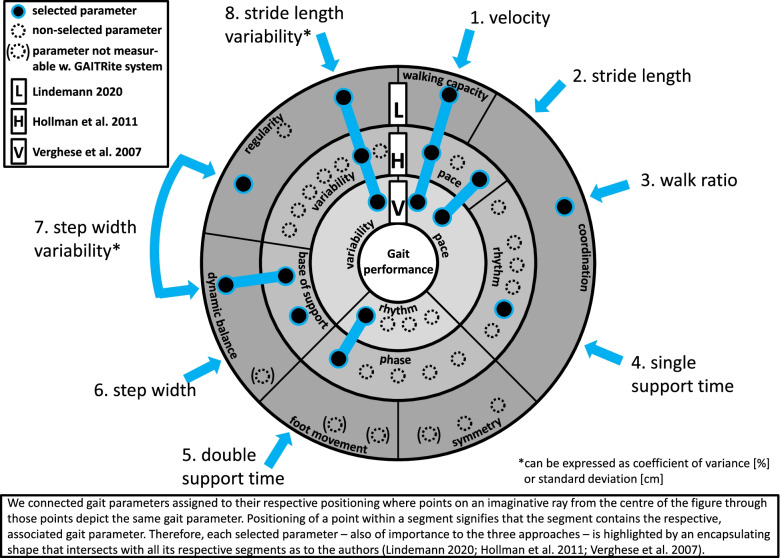
Selection of gait parameters across segments of gait performance for reference values based on literature

At present, to the knowledge of the authors, there exist three different frameworks that assess gait characteristics as we visualised in Fig. 1. The group of Verghese and colleagues [15] included the factors “pace” (gait speed, stride length), “rhythm” (with double support time); and “variability” (standard deviation of stride length) in their framework and was expanded by postural control and asymmetry [17]. The group of Hollman and colleagues [14] - in extension to Verghese’s three gait factors - added step width and step width CV as factors of “base of support” (termed “H-H base support” in GAITRite), and gait parameters representing “phase” (e.g. double support time). While the domains by Hollman are data driven, domains by Verghese are related to cognitive decline. In contrast, domains defined by Lindemann [13] are disease oriented and comprise “walking capacity” (gait speed), “regularity” (e.g. stride length CV and step width CV), “coordination” (walk ratio), “dynamic balance” (e.g. step width CV), “symmetry” (e.g. step length difference), and “foot movement” (e.g. foot clearance height). All mentioned frameworks categorised gait parameters into domains as visualised in Fig. 1. Due to this heterogeneity in gait characteristics Beauchet and colleagues [18] published - in the name of international gait consortium - guidelines for assessing gait and reference values for older adults and proposed which gait variables should be included as a minimum or full data set.

The preliminary work by Verghese [15], Hollman [14] and Lindemann [13] formed our framework based approach. Our extracted and reported gait characteristics fitted in Beauchets gait guidelines and were congruent in all frameworks (Fig. 1).

Deeper knowledge about reference values for gait characteristics in community-dwelling people may substantially aid patient-individualised preventative interventions. This will require methods to identify functional decline in early stages within the functional continuum in order to maintain the functional level and an independent lifestyle [1]. Especially facing the present and future staff and financial challenges of health systems, it is crucial to delay the need for geriatric treatment and nursing care [19]. Functional decline is already observable in an ambulant setting for community-dwelling older persons and has to be thoroughly understood and incorporated for treatments in ambulatory and clinical settings [1].

Methods to measure gait characteristics or gait speed range from using a simple stop watch to sophisticated data measures with wearables such as sensors and algorithm as are currently developed in an EU project as MOBILIZE-D. At present the golden standard for gait analysis is the GAITRite system but this may change in the future.

As most researchers rarely include older persons with different physical functional level and use different gait characteristics there is a lack of knowledge about gait changes in community-dwelling older persons based on their functional ability.

### Aim

Our main objective for this study was to investigate gait differences in community-dwelling older persons with different functional ability ranging from robust to transient (postrobust and prefrail) to frail physical status as observable for gait variables based on Fig. 1 using an objective gait system (GAITRite).

We wanted to compare gait changes in normal (preferred) vs fast gait condition in the robust, transient and frail older persons. Additionally, we investigated differences in sex and age for selected gait variables in preferred and fast gait condition.

## Methods

### Design

We combined baseline data from two studies to report reference values of gait characteristics in community-dwelling older persons with different physical functional levels. The first study (LASTIMO) focused on persons with many functional resources. The second study (NWGA) focused on persons with few functional resources and increasing functional risks. Inclusion criteria in LASTIMO was functional level “Robust” whereas in NWGA it was functional status “postRobust” and “preFrail” (grouped as “Transient”) and “Frail” according to the LUCAS Functional Ability Index (FAI) applied [20]. For both studies, inclusion age was 70 years and over, and study participants were recruited from the same district in Hamburg, Germany. For LASTIMO a call to participation was published in local newspapers (2016) while for NWGA health insurance companies sent invitation letters to older insured persons (2017). To receive target group specific interventions according to their individual functional level interested respondents filled in the LUCAS FAI and mailed it free of charge to the study centre where the FAI was evaluated. Eligible individuals were invited and agreed to participate in a comprehensive geriatric assessment (CGA) at the study centre of a geriatric clinic. Reported reference values of gait characteristics originated from this baseline assessment in the testing period, after provision of written consent.

In order to differentiate our sample by physical functional level we made use of two instruments that measure functional ability.

First, we used the self-administered screening tool LUCAS Functional Ability Index (LUCAS FAI) that depicts functional ability and decline across the entire spectrum from robust to frail and can be completed by older persons within 5 min. The FAI was developed using longitudinal data of the Longitudinal Urban Cohort Ageing Study (LUCAS), and provides an easy way to screen the heterogeneous population of community-dwelling senior citizens for high functional ability and early signs of functional decline [21]. The FAI is distinct from Basic Activities of Daily Living (BADL) dependency [22]. It incorporates Fried’s phenotype frailty criteria [23] but also functional resources focusing on good endurance, frequent outside walking, moderate and strenuous sports or recreation, regular volunteer work, and no limitation of activity due to fear of falling which may help to compensate functional losses. The FAI discriminates between four functional classes (Robust, postRobust, preFrail and Frail), i.e. the FAI is distinct from multiple scales that also assess frailty and count deficits in health, but do not incorporate health risks and health resources to equal degree [24, 25]. Therefore, the term” Frail” used with respect to the FAI is, by definition, broader when compared to the conventional view [26]. The FAI predicted long-term changes in functional status, future need of nursing care and mortality and is detailed elsewhere [20].

Second, in addition to the FAI, we conducted the Short Physical Performance Battery (SPPB), a standardised, widely established performance instrument to measuring lower extremity function in clinical and research settings [27, 28]. The SPPB comprises three subtests which assess balance (in side-by side, semi-tandem and full-tandem position), short distance walking speed (4 m gait speed test), and the ability to repeatedly rise from and sit down on a chair (chair stand test). Each component scores 0 to 4 points resulting in a combined SPPB score ranging between 0 and 12 points. Total performance time, when guided by a trained instructor ranges between 10 and 15 minutes [27]. The SPPB score predicts disability and all-cause mortality in community-dwelling older persons, and lower scores may define frailty [29, 30]. The functional ability of participants may be categorised as robust (SPPB score of 11 to 12), transient (8 to 10) or frail (0 to 7) denoting high, moderate and low physical performance as suggested in the literature [30–32]. These categories correspond to the LUCAS FAI groups Robust, Transient (postRobust and preFrail) and Frail respectively [20].

### Sample

The gait parameters of study participants were evaluated from combined data of two studies assessing community-dwelling persons aged 70 years and over with different physical functional level (without need of nursing care) according to the LUCAS FAI [20] in one district in Hamburg, Germany: LASTIMO study (*n* = 104 Robust persons) and NWGA study (*n* = 635 non-Robust persons), of which 97 were excluded due to use of mobility aids e.g. canes, crutches, walkers or wheelchairs, resulting in a total of *n* = 642 participants (*n* = 104 Robust, *n* = 323 Transient *n* = 215 Frail). In order to assure statistical stability in analyses we combined the transitioning groups with decline in resources and increase in risk factors (postRobust and preFrail) into a group termed Transient [20]. Details see flow chart (Fig. S1: Supplementary Fig. 1).

### Ethics

Eligible participants provided written informed consent and agreed upon the use of pseudonymous data for analysis. Study recruitment, procedure and all personal data used were approved by the Ethics Committee of the General Medical Council Hamburg: LASTIMO (PV5179) in 2016, NWGA (PV5484) in 2017 and in accordance with the principles of the Declaration of Helsinki, the rules of the German Personal Data Protection Act and the Hamburg Data Protection Act.

### Assessment

We characterised our study population with self-reported data on socio-demographics, medical conditions and data recorded with instruments performed in the CGA at baseline. These included chronological age, sex, falls (“During the 12 past months, have you ever fallen to the ground or floor?” yes/no) and fear of falling (“Do you limit your activities because you are afraid you will fall?” yes/no), heart disease as an example of chronic condition (“Have you ever had coronary heart disease, heart attack, heart rhythm disturbance, dyspnoea, angina pectoris or cardiac syncope?” yes/no), the 9-question Patient Health Questionnaire (PHQ-9), a validated measure for detecting depression [33] from the German version [34] and the question for chronic pain (“Do you have pain that never completely goes away? “yes/no) from the Behavioural Rating Scale (BRS-6) [35]. Additional characteristics have been measured with standardised instruments: The Clock Completion Test (CCT) [36], Timed Up and Go test (TUG) [37], Body Mass Index (BMI) and the Short Physical Performance Battery (SPPB) [27].

Quantifying spatio-temporal gait parameters have been measured using an electronic walkway (hardware: GAITRite® 610 @120 Hz; software: Platinum v.5.8.5) with 610 cm of active pressure sensor pads that records over 50 gait parameters. Setting, setup and testing protocol was in accordance with the standard of the European GAITRite Network Group [38]. The validity of the GAITRite system has been shown previously [39]. For each participant leg length left and leg length right were manually measured in cm and entered in the GAITRite system before starting the walks on the electronic system to comply with the requirements to normalise all gait parameters. In addition, height and weight of each participant were manually measured and entered in the GAITRite system. Each participant performed one walk for each condition after a practice trial for familiarisation to the GAITRite system.

We selected the following eight parameters based on literature as depicted in Fig. 1:

1. Velocity [cm/s]: Ratio of distance walked in centimetres divided by time elapsed in seconds.
2. Stride length [cm]: Distance in centimetres between the heel points of two consecutive footprints of the same foot on the line of progression (Fig. S2: Supplementary Fig. 2: S_1_L).
3. Walk ratio [cm/(steps/min)]: Ratio of step length in centimetres by step frequency in steps per minute.
4. Single support time [ms]: Period in milliseconds during the gait cycle when only one foot touches the ground.
5. Double support time [ms]: Period in milliseconds during the gait cycle when both feet touch the ground at the same time.
6. Step width [cm]: Perpendicular distance in centimetres between midline midpoint of one footprint and the line of progression formed by the midline midpoints of the previous and following footprint which are both footprints of the opposite foot (Supplementary Fig.S 2: S_2_W)
7. Step width variability [%]: Variability of step width expressed as coefficient of variance (CV) in percent applying the formula: (standard deviation/mean)*100.
8. Stride length variability [%]: Variability of stride length expressed as coefficient of variance (CV) in percent applying the formula: (standard deviation/mean)*100.

The selected gait parameters cover all segments of the approaches (Fig. 1) except for symmetry and foot movement where the latter cannot be measured by the applied gait analysis system. Although gait symmetry can be measured with the GAITRite system we did not include it with our selected variables, as symmetry is mostly used in disease-oriented approaches. Moreover, seven of the eight parameters were recommended in guidelines for assessment of gait and reference values for spatiotemporal gait parameters in older adults by Beauchet and colleagues [18]. We report reference values of gait characteristics from walks at normal (preferred) and at fast speed (participants were instructed to walk as fast as possible without running), where gait speed is reported in cm/s.

### Data analysis

Statistical analyses were performed on pseudonymous data in SPSS v.25 and Stata v.15. For univariate analyses as presented in Tables 2, 3, 4, 5, 6, 7, 8, 9 and supplementary tables 1–6, we used descriptive methods, Chi^2^-tests and t-tests; when data was not normally distributed we used the Welch test. For multivariate analyses we applied one-way ANOVAs; when data was not normally distributed we used the Chi^2^-test. *P*-values < 0.05 were considered significant, *p* values < 0.001 as highly significant. Tables and figures were generated with MS Excel and MS PowerPoint.

To the knowledge of the authors no such gait analysis has been done before. As the large sample size of both studies provided a sufficient data basis for this analysis, no power calculation was done a priori.

## Results

### Description of the sample

From 642 participants (age 78.5 ± 4.8; *n* = 233 male, *n* = 409 female) 48.1% had fear of falling and 36.1% fell within the last 12 months. The BMI was 27.3 ± 4.4 kg/m^2^. Heart disease was reported for 51.9% of participants, and 51.2% of the sample suffered from chronic pain. PHQ-9 showed little to no indication for depression (3.1 ± 3.2 points) and CCT little to no cognitive impairment (1.9 ± 0.9 points). Physical functioning by TUG was 11.9 ± 3.2 s and categorisations by SPPB were: robust (11–12 points) 27.1%, transient (8–10 points) 44.2% and frail (0–7 points) 28.7%, and by LUCAS FAI groups respectively: Robust 16.2%, Transient 50.3% and Frail 33.5% (Table 1).

**Table 1:** Characteristics (*N* = 642)

**Characteristics (self-reported)**	**Expression**	**n (%) or mean ± SD (range min - max)**
Group size	Number	642
Sex	male / female	233 (36.3) / 409 (63.7)
Age	Mean ± SD	78.5 ± 4.8 (range 70.1–93.4)
Fear of falling	Yes	309/631 (48.1)
Falls during last 12 months	Yes	232/640 (36.1)
Patient Health Questionnaire German version (PHQ-9)	Mean ± SD (continuous score)	3.1 ± 3.2 (range 0–24)
Clock completion test (CCT)	Mean ± SD (Shulman)	1.9 ± 0.9 (range 1–5)
Body mass index (BMI) [kg/m^2^]	Mean ± SD	27.3 ± 4.4
Heart disease	Yes	333/622 (51.9)
Timed Up and Go (TUG) [s]	Mean ± SD	11.9 ± 3.2 (range 6.1–32.2)
Pain: 6-point Behavioral Rating Scale (BRS-6)	Yes	329/641 (51.2)
Short Physical Performance Battery (SPPB)^a^	12–11 points: Robust	174/642 (27.1)
	10–8 points: Transient	284/642 (44.2)
	7–0 points: Frail	184/642 (28.7)
Functional competence according to LUCAS Functional Ability Index^b^	Robust	104/642 (16.2)
	Transient	323/642 (50.3)
	Frail	215/642 (33.5)

*SD* Standard deviation, *n* Number of cases

^a^Classification on own clinical experience and on Roquebert and colleagues [31], Cesari and colleagues [32] and Vasunilashorn and colleagues [40]

^b^Classification according to LUCAS Functional Ability Index [20]

### Differences between gait characteristics between the three physical functional levels

For all subsequent results, reported values were grouped where the first entries correspond to stratification by SPPB (see Table 2) whereas the second entries referred to stratification by LUCAS FAI (see Table 3). As aforementioned, the categories SPPB robust, transient and frail can be interpreted as high, moderate and low physical performance respectively. Analogously, Tables 4 and 5 show reference values for fast gait speed condition.

1. *Velocity:* Reporting grouped values for walks at preferred gait speed by SPPB/LUCAS FAI, robust walked at 129.9 cm/s (SPPB) vs 133.9 cm/s (FAI), transient at 112.8 cm/s (SPPB) vs 112.2 cm/s (FAI) and frail at 89.2 cm/s (SPPB) vs 97.1 cm/s (FAI) on average respectively (Tables 2 and 3). At fast gait speed we measured 172.4 cm/s (SPPB) vs 176.6 cm/s (FAI) for robust, 152.5 cm/s (SPPB) vs 151.4 cm/s (FAI) for transient and 123.8 cm/s (SPPB) vs 134.4 cm/s (FAI) for frail (Tables 4 and 5). Overall, gait speed decreased alongside functional decline (SPPB and FAI) showing highly significant differences (*p* < 0.001) in all tests (Tables 2, 3, 4 and 5).
2. *Stride length:* decreased from (SPPB vs FAI) 134.8 cm vs 139.0 cm (robust) over 122.8 cm vs 121.7 cm (transient) to 102.8 cm vs 109.2 cm (frail) at preferred speed (Tables 2 and 3); respectively for fast gait speed: 152.2 cm vs cm (robust), 140.0 cm vs 138.9 cm (transient) and 121.6 cm vs 128.4 cm (frail) (Tables 4 and 5). Stride length decreased alongside functional decline where differences were highly significant for all comparisons (*p* < 0.001) for both, SPPB and FAI, at preferred and fast gait speed.
3. *Walk ratio:* Generally, walk ratio decreased alongside functional decline (SPPB and FAI) showing significant differences in all tests ranging between *p* < 0.001 and *p* = 0.016 for preferred and fast gait speed (Tables 2, 3, 4 and 5). At preferred gait speed walk ratio was minimal for frail at 0.50 cm/(step/min) in SPPB vs 0.52 cm/(step/min) in FAI and maximal for robust at 0.59 cm/(step/min) in SPPB vs 0.60 cm/(step/min) in FAI (Tables 2 and 3). Similarly, but slightly higher walk ratios were measured for fast gait speed condition (Tables 4 and 5)
4. *Single support time:* At preferred gait speed, single support time increased minimally by milliseconds from robust to frail for both, SPPB and FAI, showing significant differences in all comparisons by SPPB categorisation except for between transient and frail. Differences were not significant for categorisation by FAI (Tables 2 and 3). At fast gait speed, significant differences showed for SPPB except for between robust and transient. For FAI at fast gait speed no significant differences existed aside from between robust and frail (Tables 4 and 5).
5. *Double support time:* Double support time showed highly significant differences (*p* < 0.001) at preferred and fast gait speed for both instruments (SPPB, FAI) over all categorisations by functional level (robust, transient, frail). The robust group walked at 260 ms vs 255 ms, the transient at 304 ms vs 305 ms and the frail group at 364 ms vs 342 ms at preferred gait speed condition (Tables 2 and 3). At fast gait speed, we measured 182 ms vs 178 ms for robust, 216 ms vs 216 ms for transient and 263 ms vs 246 ms for frail (Tables 4 and 5).
6. *Step width:* increased alongside functional decline where differences were mostly significant at preferred gait speed (SPPB, FAI) and fast gait speed (SPPB) but never for FAI at fast gait speed. At preferred gait speed, step width values increased from robust 8.9 cm (SPPB) vs 9.4 cm (FAI) over 9.7 cm (SPPB) vs 9.7 cm (FAI) at transient to 11.6 cm (SPPB) vs 10.8 cm (FAI) at frail (Tables 2 and 3). At fast gait speed, we also saw an increase from robust over transient to frail in SPPB and FAI featuring slightly lower values compared to preferred gait speed (Tables 4 and 5).
7. *Step width CV:* was never significant for either SPPB or FAI at preferred gait speed; and only significant between robust and transient for fast gait speed. Values always peaked for transient (SPPB, FAI) at both speed conditions, whereas step width CV was generally the lowest for frail (SPPB, FAI) with the exception of FAI at fast gait speed (Tables 2, 3, 4 and 5).
8. *Stride length CV:* Differences were highly significant (*p* < 0.001) alongside functional decline at preferred and fast gait speed in both SPPB and FAI with the minor exception between transient and frail for FAI at fast gait speed. At preferred gait speed, stride length CV increased from (SPPB vs FAI) 3.2% vs 2.9% at robust, 3.8% vs 4.0% at transient to 5.4% vs 4.8% at frail (Tables 2 and 3). At fast gait speed we measured 2.8% vs 2.6% for robust, 3.6% vs 3.6% for transient and 4.2% vs 4.0% for frail with a mean increase of about 0.7% vs 0.7% per transition (Tables 4 and 5).

**Table 2:** Gait parameters at preferred speed by Short Physical Performance Battery (SPPB) score

**Pref *n* = 642**				***p*-value**			
**Functional ability**	**SPPB 12–11** Robust	**SPPB 10–8** **Transient**	**SPPB 7–0** Frail	**total**	**R/T**	**R/F**	**T/F**
	*n* = 174	*n* = 284	*n* = 184				
**Gait Parameter**							
1. Velocity [cm/s]	129.9 ± 18.0	112.8 ± 18.2	89.2 ± 19.2	**< 0.001**	**< 0.001**	**< 0.001**	**< 0.001**
2. Stride length [cm]	134.8 ± 15.8	122.8 ± 15.4	102.8 ± 17.4	**< 0.001**	**< 0.001**	**< 0.001**	**< 0.001**
3. Walk ratio^a^ [cm/(steps/min)]	0.59 ± 0.08	0.56 ± 0.08	0.50 ± 0.08	**< 0.001**	**0.002**	**< 0.001**	**< 0.001**
4. Single support time [ms]	392 ± 28	398 ± 33	402 ± 39	**0.014**	**0.032**	**0.004**	0.266
5. Double support time [ms]	260 ± 43	304 ± 56	364 ± 76	**< 0.001**	**< 0.001**	**< 0.001**	**< 0.001**
6. Step width^b^ [cm]	8.9 ± 2.9	9.7 ± 3.0	11.6 ± 3.9	**< 0.001**	**0.007**	**< 0.001**	**< 0.001**
7. Step width^b^ CV^c^ [%]	25.3 ± 16.0	26.2 ± 14.8	23.7 ± 14.8	0.216	0.535	0.327	0.073
8. Stride length CV^c^ [%]	3.2 ± 1.5	3.8 ± 1.8	5.4 ± 3.0	**< 0.001**	**< 0.001**	**< 0.001**	**< 0.001**

*R* ROBUST, *T* TRANSIENT, *F* FRAIL

^a^formula: (Stride Length / 2) / (Number of Steps/min)

^b^the GAITRite system uses the term “Heel to Heel Base of Support”

^c^coefficient of variance; formula: (Standard Deviation / Mean) * 100

**Table 3:** Gait parameters at preferred speed by LUCAS Functional Ability Index (LUCAS FAI)

**Pref *n = 642***				***p*-value**			
**Functional ability**	**LUCAS FAI** **Robust**	**LUCAS FAI** **Transient**	**LUCAS FAI** **Frail**	**total**	**R/T**	**R/F**	**T/F**
	*n* = 104	*n* = 323	*n* = 215				
**Gait Parameter**							
1. Velocity [cm/s]	133.9 ± 18.6	112.2 ± 20.9	97.1 ± 20.9	**< 0.001**	**< 0.001**	**< 0.001**	**< 0.001**
2. Stride length [cm]	139.0 ± 16.0	121.7 ± 17.2	109.2 ± 18.8	**< 0.001**	**< 0.001**	**< 0.001**	**< 0.001**
3. Walk ratio^a^ [cm/(steps/min)]	0.60 ± 0.08	0.55 ± 0.08	0.52 ± 0.09	**< 0.001**	**< 0.001**	**< 0.001**	**< 0.001**
4. Single support time [ms]	394 ± 27	397 ± 33	400 ± 37	0.368	0.364	0.125	0.438
5. Double support time [ms]	255 ± 46	305 ± 66	342 ± 71	**< 0.001**	**< 0.001**	**< 0.001**	**< 0.001**
6. Step width^b^ [cm]	9.4 ± 2.7	9.7 ± 3.2	10.8 ± 3.9	**< 0.001**	0.360	**< 0.001**	**0.002**
7. Step width^b^ CV^c^ [%]	24.9 ± 16.4	25.9 ± 14.3	24.3 ± 15.7	0.485	0.598	0.739	0.228
8. Stride length CV^c^ [%]	2.9 ± 1.5	4.0 ± 1.9	4.8 ± 2.9	**< 0.001**	**< 0.001**	**< 0.001**	**< 0.001**

*R* ROBUST, *T* TRANSIENT, *F* FRAIL

^a^formula: (Stride Length / 2) / (Number of Steps/min)

^b^the GAITRite system uses the term “Heel to Heel Base of Support”

^c^coefficient of variance; formula: (Standard Deviation / Mean) * 100

**Table 4:** Gait parameters at fast speed by Short Physical Performance Battery (SPPB) score

**Fast *n* = 601**				***p*-value**			
**Functional ability**	**SPPB 12–11** **Robust**	**SPPB 10–8** **Transient**	**SPPB 7–0** **Frail**	**total**	**R/T**	**R/F**	**T/F**
	*n* = 168	*n* = 272	*n* = 161				
**Gait Parameter**							
1. Velocity [cm/s]	172.4 ± 22.1	152.5 ± 23.2	123.8 ± 22.2	**< 0.001**	**< 0.001**	**< 0.001**	**< 0.001**
2. Stride length [cm]	152.2 ± 17.9	140.0 ± 17.4	121.6 ± 18.3	**< 0.001**	**< 0.001**	**< 0.001**	**< 0.001**
3. Walk ratio^a^ [cm/(steps/min)]	0.56 ± 0.09	0.54 ± 0.09	0.50 ± 0.09	**< 0.001**	**0.008**	**< 0.001**	**< 0.001**
4. Single support time [ms]	352 ± 28	355 ± 32	365 ± 33	**< 0.001**	0.208	**< 0.001**	**0.003**
5. Double support time [ms]	182 ± 37	216 ± 49	263 ± 58	**< 0.001**	**< 0.001**	**< 0.001**	**< 0.001**
6. Step width^b^ [cm]	9.0 ± 2.8	9.2 ± 2.9	10.6 ± 3.7	**< 0.001**	0.478	**< 0.001**	**< 0.001**
7. Step width^b^ CV^c^ [%]	25.7 ± 14.7	27.4 ± 17.7	24.8 ± 14.0	0.221	0.286	0.576	0.088
8. Stride length CV^c^ [%]	2.8 ± 1.7	3.6 ± 1.8	4.2 ± 2.0	**< 0.001**	**< 0.001**	**< 0.001**	**0.001**

*R* ROBUST, *T* TRANSIENT, *F* FRAIL

^a^formula: (Stride Length / 2) / (Number of Steps/min)

^b^the GAITRite system uses the term “Heel to Heel Base of Support”

^c^coefficient of variance; formula: (Standard Deviation / Mean) * 100

**Table 5:** Gait parameters at fast speed by LUCAS Functional Ability Index (LUCAS FAI)

**Fast *n* = 601**				***p*-value**			
**Functional ability**	**LUCAS FAI** **Robust**	**LUCAS FAI** **Transient**	**LUCAS FAI** **Frail**	**total**	**R/T**	**R/F**	**T/F**
	*n* = 104	*n* = 306	*n* = 191				
**Gait Parameter**							
1. Velocity [cm/s]	176.6 ± 23.5	151.4 ± 25.4	134.4 ± 26.0	**< 0.001**	**< 0.001**	**< 0.001**	**< 0.001**
2. Stride length [cm]	155.7 ± 18.1	138.9 ± 18.5	128.4 ± 20.3	**< 0.001**	**< 0.001**	**< 0.001**	**< 0.001**
3. Walk ratio^a^ [cm/(steps/min)]	0.57 ± 0.09	0.54 ± 0.09	0.52 ± 0.09	**< 0.001**	**< 0.001**	**< 0.001**	**0.016**
4. Single support time [ms]	353 ± 27	356 ± 32	361 ± 33	0.075	0.473	**0.040**	0.072
5. Double support time [ms]	178 ± 41	216 ± 53	246 ± 56	**< 0.001**	**< 0.001**	**< 0.001**	**< 0.001**
6. Step width^b^ [cm]	9.2 ± 2.7	9.4 ± 3.0	9.9 ± 3.6	0.131	0.573	0.067	0.116
7. Step width^b^ CV^c^ [%]	22.9 ± 12.1	27.5 ± 16.2	26.1 ± 17.1	**0.039**	**0.003**	0.061	0.371
8. Stride length CV^c^ [%]	2.6 ± 1.5	3.6 ± 1.8	4.0 ± 2.0	**< 0.001**	**< 0.001**	**< 0.001**	**0.010**

*R* ROBUST, *T* TRANSIENT, *F* FRAIL

^a^formula: (Stride Length / 2) / (Number of Steps/min)

^b^the GAITRite system uses the term “Heel to Heel Base of Support”

^c^coefficient of variance; formula: (Standard Deviation / Mean) * 100

### Differences in gait characteristics based on physical functional level, sex and age

Supplementary Table 1a-d (Table S1) shows the same data based on functional level and further subdivided by sex and age. Further distinguishing subgroups of sex and age by functional ability level revealed higher ranges for values of gait parameters: For example, at preferred gait speed velocity of women in the age group of 70–79 years ranged between 92.3 cm/s (SPPB frail) and 131.3 cm/s (SPPB robust) (Table S1: Supplementary Table 1a), whereas women 80 years and older walked at between 87.8 cm/s (SPPB frail) and 117.8 cm/s (SPPB robust) at preferred gait speed (Table S1: Supplementary Table 1c). A similar observation also showed for the categorisation by LUCAS FAI (Table S1: Supplementary Table 1b + d). These considerable variances from the mean within one of the same age group but different functional levels are depicted in Supplementary Fig. 3 (Fig. S3) for the example of velocities of women at preferred gait speed.

At preferred gait speed, the subgroup analysis differentiated by functional level and sex only consistently showed significant differences across all functional ability level comparisons by both, SPPB and LUCAS-FAI, for the following five of eight gait parameters: Velocity, stride length, walk ratio, double support time and stride length CV (Table S2: Supplementary Table 2a + b).

At fast gait speed the consistent differences remained for functional ability level comparisons by SPPB, whereas for functional ability level comparisons by LUCAS FAI differences also showed for velocity, stride length and double support time but not for walk ratio and stride length CV (Table S3: Supplementary Table 3a + b).

The subgroup analysis for functional level and age, at preferred gait speed, consistently showed significant differences across all functional ability level comparisons by both, SPPB and LUCAS FAI, for only the following three of eight gait parameters: Velocity, stride length and double support time (Table S4: Supplementary Table 4a + b).

At fast gait speed, the consistent differences remained for functional ability level comparisons by SPPB, whereas for functional ability level comparisons by LUCAS FAI differences were lost for the gait parameter stride length CV, and all comparisons between Robust and Transient became non-significant within the age group of 80 years and older for all gait parameters (Table S5: Supplementary Table 5a + b).

For analogous comparison at fast gait speed see Supplementary Table 6a + b + c + d (Table S6).

In summary, Tables 6, 7, 8, and 9 compress all gait parameters at preferred speed with constantly significant differences (*p* < 0.001) between the functional classes Robust, Transient, Frail by functional ability (SPPB, LUCAS FAI), sex (male, female) and age groups (70–79, 80+).

**Table 6:** Functional classes by Short Physical Performance Battery (SPPB) score and age groups 70–79 years

**Gait Parameter**	**Male**				**Female**			
**Functional ability**	**70-79y** **Total**	**70-79y** **Robust**	**70-79y** **Transient**	**70-79y** **Frail**	**70-79y** **Total**	**70-79y** **Robust**	**70-79y** **Transient**	**70-79y** **Frail**
	*n* = 149	*n* = 53	*n* = 68	*n* = 28	*n* = 259	*n* = 86	*n* = 121	*n* = 52
**Pref** ***n*** **= 408**								
1. Velocity [cm/s]	117.4 ± 24.6	133.6 ± 19.0	115.9 ± 19.2	90.8 ± 21.6	115.9 ± 22.5	131.3 ± 16.4	115.1 ± 18.1	92.3 ± 19.4
2. Stride length [cm]	130.8 ± 21.9	144.1 ± 15.2	130.2 ± 17.3	107.2 ± 22.7	122.3 ± 16.9	132.3 ± 13.4	122.5 ± 13.4	105.3 ± 16.1
3. Walk ratio^a^ [cm/(steps/min)]	0.61 ± 0.10	0.65 ± 0.07	0.61 ± 0.09	0.53 ± 0.11	0.54 ± 0.07	0.56 ± 0.06	0.55 ± 0.06	0.50 ± 0.08
5. Double support time [ms]	312 ± 72	273 ± 46	317 ± 58	375 ± 94	291 ± 66	248 ± 38	291 ± 50	361 ± 75
8. Stride length CV^b^ [%]	3.8 ± 2.0	3.1 ± 1.3	3.7 ± 2.1	5.3 ± 2.1	3.6 ± 1.8	3.1 ± 1.6	3.5 ± 1.6	4.6 ± 2.2

^a^formula: (Stride Length / 2) / (Number of Steps/min)

^b^coefficient of variance; formula: (Standard Deviation / Mean) * 100

**Table 7:** Functional classes by LUCAS Functional Ability Index (LUCAS FAI) and age groups 70–79 years

**Gait Parameter**	**Male**				**Female**			
**Functional ability**	**70-79y** **Total**	**70-79y** **Robust**	**70-79y** **Transient**	**70-79y** **Frail**	**70-79y** **Total**	**70-79y** **Robust**	**70-79y** **Transient**	**70-79y** **Frail**
	*n* = 149	*n* = 40	*n* = 65	*n* = 44	*n* = 259	*n* = 51	*n* = 138	*n* = 70
**Pref** ***n*** **= 408**								
1. Velocity [cm/s]	117.4 ± 24.6	138.3 ± 17.0	114.4 ± 21.8	103.0 ± 22.1	115.9 ± 22.5	134.1 ± 17.5	116.0 ± 19.9	102.4 ± 21.5
2. Stride length [cm]	130.8 ± 21.9	147.9 ± 13.7	128.8 ± 19.9	118.2 ± 21.3	122.3 ± 16.9	135.0 ± 12.9	122.4 ± 15.0	133.0 ± 17.2
3. Walk ratio^a^ [cm/(steps/min)]	0.61 ± 0.10	0.66 ± 0.07	0.61 ± 0.09	0.57 ± 0.11	0.54 ± 0.07	0.57 ± 0.06	0.54 ± 0.06	0.52 ± 0.07
5. Double support time [ms]	312 ± 72	265 ± 43	318 ± 65	348 ± 80	291 ± 66	240 ± 43	291 ± 58	328 ± 70
8. Stride length CV^b^ [%]	3.8 ± 2.0	2.6 ± 1.2	4.3 ± 2.1	4.2 ± 1.9	3.6 ± 1.8	2.9 ± 1.6	3.6 ± 1.6	4.0 ± 2.1

^a^formula: (Stride Length / 2) / (Number of Steps/min)

^b^coefficient of variance; formula: (Standard Deviation / Mean) * 100

**Table 8:** Functional classes by Short Physical Performance Battery (SPPB) score and age groups 80 years and over

**Gait Parameter**	**Male**				**Female**			
**Functional ability**	**80y+** **Total**	**80y+** **Robust**	**80y+** **Transient**	**80y+** **Frail**	**80**y**+** **Total**	**80y+** **Robust**	**80y+** **Transient**	**80y+** **Frail**
	*n* = 84	*n* = 14	*n* = 36	*n* = 34	*n* = 150	*n* = 21	*n* = 59	*n* = 70
**Pref** ***n*** **= 234**								
1. Velocity [cm/s]	102.2 ± 23.9	125.5 ± 18.7	108.2 ± 19.0	86.3 ± 19.6	99.7 ± 20.2	117.8 ± 16.9	107.2 ± 14.9	87.8 ± 17.8
2. Stride length [cm]	117.0 ± 21.2	134.5 ± 16.9	123.9 ± 17.2	102.4 ± 17.3	108.4 ± 16.1	121.8 ± 12.9	114.3 ± 11.0	99.3 ± 15.6
3. Walk ratio^a^ [cm/(steps/min)]	0.56 ± 0.09	0.60 ± 0.09	0.60 ± 0.09	0.51 ± 0.08	0.49 ± 0.07	0.53 ± 0.05	0.51 ± 0.06	0.47 ± 0.07
5. Double support time [ms]	343 ± 74	278 ± 43	333 ± 71	380 ± 66	318 ± 68	263 ± 36	296 ± 47	353 ± 73
8. Stride length CV^b^ [%]	5.0 ± 2.8	3.8 ± 1.9	4.5 ± 2.1	6.0 ± 3.5	4.7 ± 2.8	3.4 ± 1.3	4.0 ± 1.8	5.7 ± 3.5

^a^formula: (Stride Length / 2) / (Number of Steps/min)

^b^coefficient of variance; formula: (Standard Deviation / Mean) * 100

**Table 9:** Functional classes by LUCAS Functional Ability Index (LUCAS FAI) and age groups 80 years and over

**Gait Parameter**	**Male**				**Female**			
**Functional ability**	**80y+ ** **Total**	**80y+ ** **Robust**	**80y+ ** **Transient**	**80y+ ** **Frail**	**80y+ ** **Total**	**80y+ ** **Robust**	**80y+ ** **Transient**	**80y+ ** **Frail**
	*n* = 84	*n* = 6	*n* = 50	*n* = 28	*n* = 150	*n* = 7	*n* = 70	*n* = 73
**Pref** ***n*** **= 234**								
1. Velocity [cm/s]	102.2 ± 23.9	131.4 ± 18.0	104.9 ± 23.1	91.2 ± 19.9	99.7 ± 20.2	109.8 ± 21.3	107.9 ± 18.6	90.8 ± 17.8
2. Stride length [cm]	117.0 ± 21.2	143.2 ± 10.2	120.2 ± 19.1	105.7 ± 19.9	108.4 ± 16.1	114.1 ± 16.4	114.9 ± 14.7	101.5 ± 14.7
3. Walk ratio^a^ [cm/(steps/min)]	0.56 ± 0.09	0.65 ± 0.04	0.58 ± 0.08	0.51 ± 0.10	0.49 ± 0.07	0.49 ± 0.05	0.51 ± 0.06	0.48 ± 0.07
5. Double support time [ms]	343 ± 74	276 ± 51	338 ± 75	365 ± 65	318 ± 68	290 ± 51	295 ± 63	342 ± 67
8. Stride length CV^b^ [%]	5.0 ± 2.8	3.5 ± 1.8	4.3 ± 2.1	6.5 ± 3.5	4.7 ± 2.8	3.6 ± 1.0	4.2 ± 2.0	5.3 ± 3.5

^a^formula: (Stride Length / 2) / (Number of Steps/min)

^b^coefficient of variance; formula: (Standard Deviation / Mean) * 100

## Discussion

The main aim of our study was to report on characteristics of different gait variables based on a theoretically derived approach in older community-dwelling persons with different physical functional levels. The novelty of our gait analyses is that gait is related to function which is becoming much more important in the geriatric field of health prevention. Often gait data is related to age, gender or disease but far more rarely to function. Gait variables were obtained with an objective gait analysis system. Physical functional level was defined in two different ways, SPPB (measured in CGA) and LUCAS questionnaire (self-administered), showing high congruency in forming three different physical functional levels ranging across robust, transient and frail older community-dwelling persons. We thereby adopted the approach of the WHO according to which ageing and health incorporate functional ability as a main category [1].

This work focuses on showing a carefully selected subset of meaningful gait parameters that originate from distinct segments of gait (Fig. 1) for community-dwelling older people living at home with regard to functional ability levels. Therefore, we do not debate on gait parameters by sex or age in detail. Nevertheless, sex and age differences are discussed in the context of functional ability levels.

Additionally, our goal is to investigate differences in preferred and fast gait speed conditions based on the three different physical functional levels – especially with respect to early identification of functional decline.

Our data in Table 1 demonstrate that we included the “typical” older community-dwelling person with respect to age range (70.1 years to 93.4 years), and present common demographical distribution between the sexes in the Western world (63.7% females,36.3% males). The low depression score (PHQ-9) and the reported rates of chronic pain (51.2%) and heart disease (51.9%) also underline the broad range of physical and mental health conditions in our older community-dwelling sample. Also, the percentage of participants reporting a fall 12 months prior to study start (36.1%) coincides with most reported numbers (e.g. [41]), and is visible in the 48.1% reporting fear of falling.

Overall our results showed that by mere distinction by functional level a wide spectrum of functional decline can be seen for all investigated gait variables (Tables 2, 3, 4, & 5). Internal analyses showed that when differentiating by chronological age instead of functional level, the spectrum becomes noisier and loses discriminatory power. Additionally, Supplementary Tables 1a-d (Table S1), Supplementary Table 7 (Table S7) and Supplementary Fig. 3 (Fig. S3) show the importance of considering the functional level in order to unveil and better differentiate people of the same age ranges but different functional levels.

The main takeaway from Supplementary Fig. 3 (Fig. S3) is that although evaluation by chronological age is common practice, functional ability seems to be having an even larger influence on gait speed as represented with the large ranges that needs to be considered (deviations from the bold axis): Two randomly chosen older person between 70 and 79 but very different functional ability can be different from one another to a much higher degree than two older persons with an age gap of 9 years (70 vs 79) if they both feature a similar functional ability. Not considering the functional ability results in looking at gait speed means that mask the actual performance and diversity of participants within the same age group.

While many researchers and clinicians are familiar with properties of a certain functional subgroup, a broad view and understanding of the full spectrum of functional levels is desirable in order to more comprehensively understand functional decline at old age. Evidently, approaches that do not incorporate categorisations by functional levels miss out on discriminatory power useful for interventions. Therefore, in the context of studies or intervention programs one may wrongly generalise all adults aged 70 to 79 to be less functionally restricted than adults of higher age, although this is not the case in general. Analogously, we found that adults of 80 years and above can also be robust, performing about as well as adults who are 10 years younger (Table S1: Supplementary Tables 1a-d).

In the next section we discussed our findings with regard to each individual parameter as depicted in Fig. 1.

### Velocity

As mentioned above, gait velocity – often termed gait speed – has been accepted as the “sixth vital sign” in geriatrics [5]. Velocity at < 1.0 m/s has been used for categorising high risk and at > = 1.0 m/s for low risk of health-related outcomes e.g. hospitalisation [9, 42]. Differences of 5 cm/s are associated with small and 10 cm/s with moderate effect sizes in terms of meaningful changes in gait speed [43, 44].

In our study we found that the range of velocity at preferred gait speed was similar to that commonly found in population based studies [9, 14, 45]. Comparing our data by sex and age to the results of “healthy” participants as assessed by Hollman and colleagues [14], the velocity from age range 70 to 79 was similar (− 0.4 to 5.0% difference), at age 80 and above it was slightly different (− 1.3% to − 8.8%). As Hollmann did not categorise by function and since for a larger part of our population functional decline has already set in, this could explain discrepancies. Furthermore, our results of frail participants are similar to results of the frail group in Verghese & Xue [46].

With regard to fast gait speed, groups of older participants in Almarwani and colleagues [47] showed similar gait speeds in fast gait speed condition when compared with robust participants from our study. Furthermore, the gait speed of the speed-matched older group was quite similar to the robust group when comparing preferred gait speeds.

### Stride length

Reduced stride length is one kinematic change that manifests in “cautious gait” among other gait parameters such as increased double support time and heel and toe related parameters [48, 49]. Stride length is associated with executive function and increased fall risk [15]. Nevertheless, one has to keep in mind that stride length depends on gait velocity [48].

### Walk ratio

Walk ratio is a reproducible [50] and gait speed invariant [51] gait parameter. It can be used to aid in diagnosis of various diseases (e.g. [52, 53]) since, as stated by Lindemann [13], and Giladi and colleagues [54] changes in walk ratio are not caused by the ageing process itself, but rather by underlying pathology.

Greater reductions in walk ratio when changing from preferred gait speed to fast gait speed were found to be associated with the risk of multiple falls, as was the case for people with overall low walk ratio during walks with fast gait speed condition [55]. We saw progressive indications for cautious gait in accordance with other results [54] along the line of functional decline in our data which was associated with increased risk of falling [56]. Interestingly, walk ratio seemed to be especially independently associated with falling in the past year for healthy older people who showed no deterioration of gait speed [57] which suggests that early fall prevention and interventions for robust or transient older people should also assess walk ratio. Naturally, if more detailed analyses of walk ratio are warranted, adjusting for body height is highly recommended for the sake of comparability between men and women [51]. The occurrence of declining walk ratios at fast vs preferred gait speeds is in line with literature [55].

### Single support time

Single support is – with regard to motor control – more challenging than double support, as it requires a higher degree of dynamic balance. In a study by Brach and colleagues [58] it was shown that stance time was related to impairments of the central nervous system which negatively impacted the motor control system.

Similar values to our findings for single support time can be found in literature [59]. Nevertheless, one has to be cautious not to overestimate differences in single support time along our stratifications of functional decline as they rarely changed by more than 10 ms across categories robust, transient and frail, where 8 ms is the temporal resolution of the walkway system operating at 120 Hz.

### Double support time

At first glance, the slight increase in single support time along functional decline seems to be counter-intuitive. However, as for many gait parameters, interpretation in isolation of other gait parameters can be problematic because gait is a complex motor-function of interacting domains as shown previously (e.g. [14]). By natural correlation, slower gait speed, accompanying functional decline, generally induces longer support times overall. Comparing functional levels, our data showed that increases in double support time greatly exceed minimal changes found for single support time. Support times are associated with rhythm of gait and memory decline [15].

### Step width

Dynamic balance of older people can be investigated by analysing step width which captures adjustments in the lateral direction necessary for maintaining balance while walking [13, 60]. An increase in step width while slowing down during walks may indicate fear of falling [61].

In terms of step width, the “healthy” study population in Hollman and colleagues [14], closely matched our robust group. With regard to the small changes between our robust and transient group one might speculate that changes in step width occur with a certain lag after functional decline has already set in. In this line, notable changes may primarily characterise the frail older persons which, in turn, is congruent with the functional decline.

### Step width CV

Step width variability pertains to rhythm and dynamic balance or base of support respectively (Fig. 1). Based on various studies (e.g. [62]) and his own data analysis, Hollman suggests that although literature saw gait parameters of variability as a collective construct, often referred to as “variability”, step width variability could be differentiated as a separate construct termed “base of support” ( [14]; Fig. 1). For analyses measurements of step width CV hold the advantage of greater magnitude relative to their noise floor, in particular when compared to other gait parameters of stride-to-stride variability. For investigating balance step width variability is also easier to assess than stride length variability as it exhibits comparatively lower dependency on gait speed [63]. Incidentally, step width variability can also be expressed using standard deviation [64]. Generally speaking, too low and too high step width variability are both associated with future falls in older persons [49, 56, 65]. Overall, step width variability is highly dependent on integrative sensing [60] and increases with age [62] as well as with sensory deficits [66].

For preferred gait speed over the course of functional decline, our data showed highest variability for the transient group, often accompanied by a larger or smaller decrease from transient to frail which sometimes put step width variability in the frail group lower than variability found in the robust group. For walking on even surfaces without obstacles one may hypothesise performance to better when the variability is relatively low [13]. Nevertheless, people exhibiting significant functional decline may show just as low or lower variability because of physical restrictions and deficits in the freedom of movement and lower gait speed and higher cadence influence the measurement. Furthermore, the transient group may experience first physical challenges where walking is in a period of adaptation of finding a new equilibrium for efficient and safe walking patterns. Moreover, despite different methodologies to assess frailty as described in literature, the process of transitioning between states has been shown to be a dynamic process [67, 68]. Finally, it is suggested that step width variability cannot differentiate between fit and frail older adults [69]. However, the given definition and selection of their study population differed from our notion of frailty and operationalisation.

In the fast gait speed condition, step width CV increased as balance was challenged. This effect was stronger for the frail group (4% higher when compared to preferred gait speed). This became especially evident when putting it into context with step width measured in centimeters: At fast gait speed, when compared to preferred gait speed, step width decreased overall, whereas step width for robust people remained at about the same value; the values of the transient group dropped slightly, values of the frail group mostly by more than 1 cm. This observation when changing speed conditions might be interpreted as robust people using resources to compensate, as first difficulties that challenge the transient group and as the lacking resources of the frail group that reached a limit visible in a relatively speaking greater increase in variability (Table S1: Supplementary Table 1a + Table S6: Supplementary Table 6a).

### Stride length CV

Variability of stride length is associated with future falls as well as cognitive decline [42, 49, 56]. High but also low variability when walking on even surfaces is associated with fall history, while too low variability can indicate a lack of adaptability when walking on more challenging surfaces [42, 65]. Increase in this parameter is also associated with poor health, functional status and physical activity [70].

One might argue that in our robust group there existed enough resources on motor control with regard to stride length CV. Therefore, stride length CV may be low in normal gait speed – even if the preferred gait speed was higher when compared to the other two functional groups transient and frail.

An increase in stride length variability over the course of functional decline was partly explained by the fact that stride length is codependent on velocity which also decreases along the same progression. However, visible differences after adjusting for gait speed may indicate risk of falling or cognitive decline [15, 49, 56].

In summary, although Tables 2 and 3 for preferred gait speed condition showed slight differences based on the underlying instruments (SPPB or LUCAS FAI) with regard to the obtained gait variables both approaches showed congruent results –which is important for future ageing research. Categorisation of older community-dwelling persons by either performed assessment (SPPB) or self-administered questionnaire (LUCAS FAI) is relevant with regard to economics (human resources in testing), research and public health-oriented prevention and health care for community-dwelling older people [1].

For preferred speed, Tables 2 and 3 demonstrated a significant decline from the robust over transient to frail group for the majority of measured gait parameters. With regard to fast gait speed (Tables 4 and 5) the difference in velocity - compared to velocity at preferred gait speed - decreased along functional decline from robust over transient to frail. This exemplified existing resources in the robust group when compared to the lack therefore in the frail group and is usually termed as walking capacity in literature [13]. Since reserve capacities can be evaluated by measuring walking capacity, this concept of capacity also conforms to the WHO report 2015 [1].

Finally, we stratified our data by three levels: functional status, sex and old age (70–79 years, Table S1: Supplementary Table 1a/b) or very old age (80 years and above, Table S1: Supplementary Table 1c/d). Interestingly, we found that conventional grouping by sex and age mask the wide continuum of values visible when taking functional status into consideration; e.g. values in the total column of Supplementary Table 1a/b (Table S1) generally represent an average similar to values in the transient column; also depicted in Supplementary Fig. 3 (Fig. S3).

For comparability with conventional stratification in literature, Supplementary Table 2a + b (Table S2) and Supplementary Table 3a + b (Table S3) showed differences by sex for preferred and fast gait speed, as well as by age. Especially Supplementary Table 4a + b (Table S4) for preferred gait speed and Supplementary Table 5a + b (Table S5) for fast gait speed (age related) underline the importance of a functional approach. As can be seen for the 70–79 years age group in Supplementary Table 4a + b (Table S4) one would miss the older community-dwelling persons “at risk” (our frail group) with gait speeds below 1.0 m/s 9. For the sake of completeness, Supplementary Table 6a-d (Table S6) showed the fast gait speed condition analogous to Supplementary Table 1a-d (Table S1). In addition, Supplementary Table 6a-d (Table S6) and Supplementary Table 1a-d (Table S1) feature a “total” column that aggregated functional groups for better comparability with existing literature.

In this regard, Beauchet and colleagues 18 report gait parameters for men and women 70 years and older. Thaler-Kal and colleagues [71] present gait parameters for men and women aged between 65 and 90 and frail/non-frail (at least one of the five Fried-Markers). In addition to this dichotomous categorisation of (non) frailty, we present reference values for gait parameters covering the broad spectrum of ageing and health as in line with the WHO 1 . Therefore, we differentiated between the groups robust, transient and frail by functional ability as measured with SPPB and LUCAS FAI. Certainly, gait parameters can be labelled and categorised by prevalence or absence of clinical pictures [71–73], yet our notion of functional ability differs from disease-oriented classifications.

Based on our results (see Tables 2, 3, 4, 5 and Table S1, Table S2, Table S3, Table S4, Table S5, Table S6: Supplementary Tables 1a-6d), Tables 6, 7, 8, and 9 demonstrates that five of the eight selected gait variables were consistently showing significant differences with regard to the functional level of the participants: gait velocity, stride length, walk ratio, double support time and stride length CV.

In the following paragraphs we highlight congruency with the literature. For walk ratio sex differences seen in our data featuring lower walk ratios for women have been reported for other populations previously [51].

With regard to fast gait speed and stride length we also found the commonly observed sex difference when subdividing by sex and functional ability level (Table S1: Supplementary Tables 1a + b). Stride length by age and sex was a few centimeters higher in robust but similar in the transient group when compared with Hollman and colleagues^14^ “healthy” people. Overall, values similar to the results of the robust and transient groups can be found in literature [59].

With regard to step width our reported differences between men and females are consistent with literature, in which men demonstrated wider steps then females (e.g. [14]).

Compared with the youngest group of healthy participants from the Mayo Clinic Study of Aging at 70–74 years of age stratified by sex [14], the group of transient participants walked at very similar double support time and standard deviations. As single support times remain stable alongside functional decline, one may suppose association with increasingly cautious gait. Further investigating percentages of gait cycle for support times confirms this trend (data not shown). We found robust males to have between 11.6% (SPPB) or 13.9% (LUCAS FAI) lower average double support times when compared to females. Our data for double support time is in the same range reported by Moe-Nilssen and Helbostad [59].

We present reference values of gait characteristics in community-dwelling older persons with different physical functional levels by means of two different validated instruments. Nevertheless, some strengths and limitations have to be acknowledged.

Strengths of the self-administered LUCAS FAI lie in the public-health based application in ambulatory setting (e.g. general practitioner). The questions of the instrument enquire performance of specific functional activities in everyday life (e.g. to take a walk outside, to perform moderate or strenuous recreational activities). Therefore, there is but marginal room for ambiguity and as much objectivity about activities factually carried out as possible is preserved. The questionnaire is easily filled in by older persons within about 5 minutes. In the long term, LUCAS FAI is highly predictive for future need of nursing care and mortality [20]. The evaluation of the LUCAS FAI aids health (care) experts by indicating a status of functional ability based on both functional resources and risks.

One strength of the SPPB is that it constitutes a well-established, standardised performance test for lower extremities performable within approximately 10–15 minutes by trained staff. In addition, the SPPB has been accepted by the EMA as an assessment tool for obtaining frailty status in older persons [30] which will increase the use of the SPPB in geriatric clinical and research routine in the future.

Data on gait from the two studies LASTIMO and NWGA were captured using the very same gait analysis system, in both, soft- and hardware. Also, the staff was the same for both studies, and so were the processes for validation thereafter: Data was screened for spatial and temporal irregularities such as overstepping boundaries of measure pads or pauses and outliers and subsequently thoroughly analysed and discussed in a panel with experts on gait and technical staff to rule out recording errors and execution errors, but all data was found to be valid.

For data consistency we used a well-established, validated computerised gait analysis system (GAITRite) for collecting reference values of gait characteristics, recording walks in compliance with the European GAITRite Network Group [38]. Presented reference values were selected in accordance with relevant segments of gait performance as identified in literature (Fig. 1).

One limitation of our findings is that we used data of two studies originally not designed for our research aim. Nevertheless, the studies were well-controlled and consistent with regards to methodology and study protocols regarding the operationalisation of gait, providing relevant data for our research question. One further limitation of our study is the cross-sectional design which precludes definite conclusions by causal explanation. In addition, a time period of about 4 to 6 weeks between the screening for functional ability (LUCAS FAI) and the comprehensive assessment including SPPB and GAITRite due to organisational constraints could have an influence on the categorisation by functional level. As we obtained not the recommended 20 gait cycles [13] with one walk for each condition on a 610 cm long GAITRite system the data on the stride length variability needs to be interpreted with caution. Nevertheless, the GAITRite system is a very reliable system, and we made great effort on the data clearance of outliers. As our aim was to present reference data, aided walks were excluded to minimize side effects caused by walking aids. Another limitation is that older community-dwelling persons with cognitive limitations were excluded, and therefore, our reference values may not be applied to groups featuring similar characteristics. A common limitation shared with other studies examining older community-dwelling people is a potential selection bias that cannot be avoided completely.

Future research needs to address the predictive values of the most important gait characteristics found in our population with regard to negative health outcomes. Another German study presented some information regarding this aspect with their cohort [71]. Another focus of future research should address community-dwelling older persons with cognitive limitations as these were excluded in our sample. For early identification and health prevention more data is needed in this specific population. Additionally, one should consider investigating the possible effects on the gait variables from exercise interventions; finally to investigate the dynamics of these variables in longitudinal studies.

#### Clinical implications

Our data showed the importance of investigating gait characteristics of the older persons stratified by functional level over stratification by age or sex. This is in line with the WHO approach for healthy ageing focusing significantly more on “function”. In clinical practice or research awareness of stratifying physical performance or research data with regard to the individual functional level is evolving in the geriatric field. In particular for community-dwelling older geriatric persons with multi-morbidity or other health issues the functional approach is gaining in importance [1, 74]. Our data showed that only five variables were showing differences with regard to the functional level of the participants. These variables of interest were gait velocity, stride length, walk ratio, double support time and stride length CV. For clinicians using in-depths gait analyses these variables should be analysed first. This is especially important as data analyses of the GAITRite system is time consuming and focussing on only these variables would be more time efficient for data analyses. Especially in primary care setting, clinicians should be aware of investigating gait based on functional level in order to identify early decline in older patients who outwardly appear to be “healthy” but in fact are not. Clinical meaningful is that the functional level was obtained with the SPPB and the LUCAS questionnaire which is a relevant aspect with regard to manpower in the assessment process.

## Conclusions

Our study showed that stratification by physical functional level rather than by sex or gender reveals a wider range of gradations. Specifically, we found the discriminative power of stratifications by SPPB to be the highest, followed by LUCAS FAI, followed by age groups and dichotomous age; where obtaining the LUCAS FAI is more cost and time effective than conducting SPPB.

Gait parameters, carefully selected by literature, showed clinically meaningful differences between the functional featuring a gradient declining from robust over transient to frail in most gait parameters.

It is not sufficient to merely focus on only one individual gait parameter such as gait speed. As true for many aspects in geriatrics, it is necessary to have a general view (multiple gait parameters) and an efficient way to record walk data at large scale. Geriatric outpatient clinics provide a setting for early, preventative identification of functional decline utilising walkway systems and deploying interventions where unsophisticated measurement by hand – when conducted without the context of a comprehensive assessment – masks crucial information required for finding proper counter-measures.

For clinical work it is important to understand the full spectrum of functional decline, not by age and sex only, but also by differences in functional levels. However, stratifications by functional levels are rarely shown. We close this gap by providing reference values that go beyond age and sex, adding the third dimension of functional ability - a spectrum best observed in community-dwelling older people. We find that for most of the carefully chosen gait parameters in this work the functional level is more determinative than is sex or age. Also, performance within an age group can substantially vary by functional ability. Therefore, clinicians are well advised to know and take functional levels into account (e.g. SPPB or LUCAS FAI).

## Supplementary Information

**Additional file 1: Supplementary Fig. 1.**

**Additional file 2: Supplementary Fig. 2.**

**Additional file 3: Supplementary Fig. 3.**

**Additional file 4: Supplementary Table 1 to Supplementary Table 7.**

## Data Availability

The datasets used and analysed during the current study are available from the corresponding author on reasonable request.

## Abbreviations

BADL: Basic Activities of Daily Living
BMI: Body Mass Index
BRS-6: Behavioural Rating Scale
CCT: Clock Completion Test
CGA: Comprehensive geriatric assessment
CV: Coefficient of variance
FAI: Functional Ability Index
LUCAS: Longitudinal Urban Cohort Ageing Study
PHQ-9: Patient Health Questionnaire (9 questions)
SPPB: Short Physical Performance Battery
TUG: Timed Up and Go test
