# Supplementary figures and images for: Reference values of gait characteristics in community-dwelling older persons with different physical functional levels
Source: BMC Geriatr. 2022 Aug 29;22:713. doi: 10.1186/s12877-022-03373-0 (PMC9422159; doi:10.1186/s12877-022-03373-0)

**Fig-S1: Supplementary Figure 1: Flow chart**

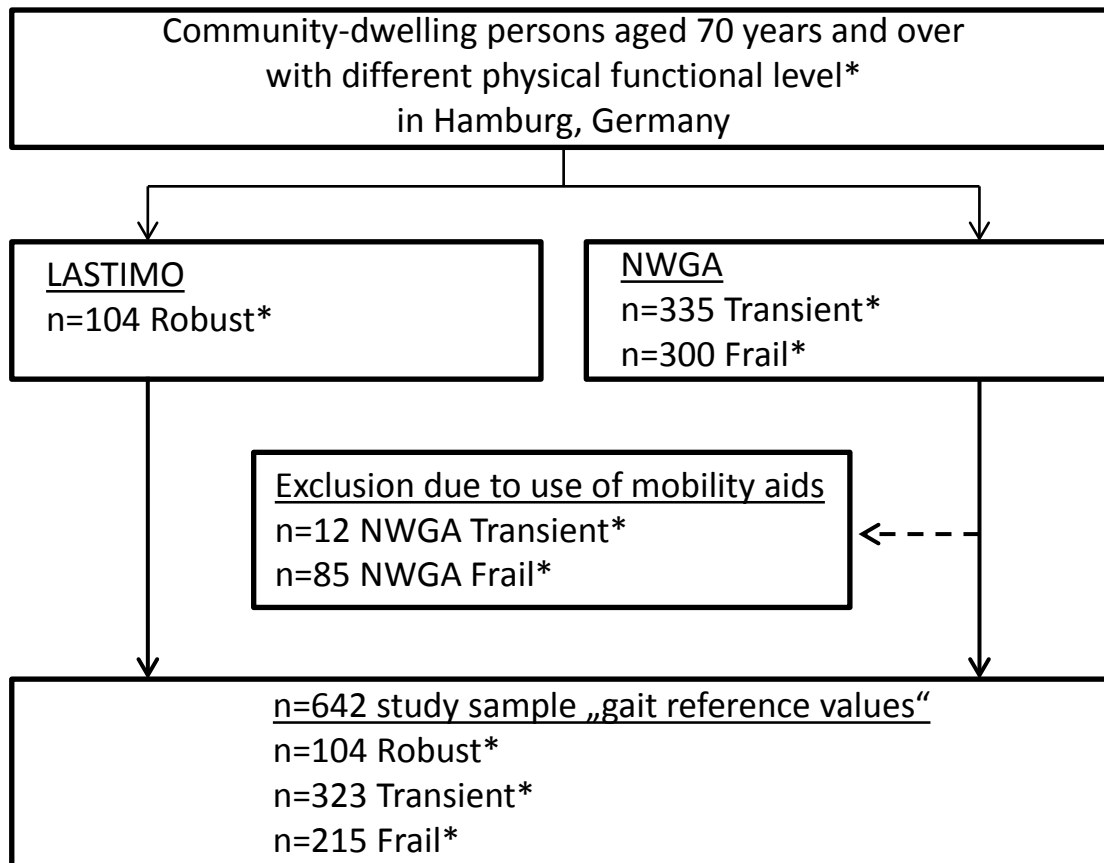

\* According to LUCAS Functional Ability Index (20)

Supplement: Supplementary file 1 — Additional file 1: Supplementary Fig. 1. [file 12877_2022_3373_MOESM1_ESM.pdf]
