# Supplementary material for: Reference values of gait characteristics in community-dwelling older persons with different physical functional levels
Source: BMC Geriatr. 2022 Aug 29;22:713. doi: 10.1186/s12877-022-03373-0 (PMC9422159; doi:10.1186/s12877-022-03373-0)
Supplement: Supplementary file 2 — Additional file 2: Supplementary Fig. 2. [file 12877_2022_3373_MOESM2_ESM.pdf]

Fig-S2: Supplementary Figure 2:
Step width and Heel-to-Heel Base of Support

While measurement in the lateral dimension of gait differs in literature, step width (**S_2W**) is one of the most prevalent terms, whereas some sources use Heel-to-Heel Base of Support (Base Width) which is the term used in the GAITRite system

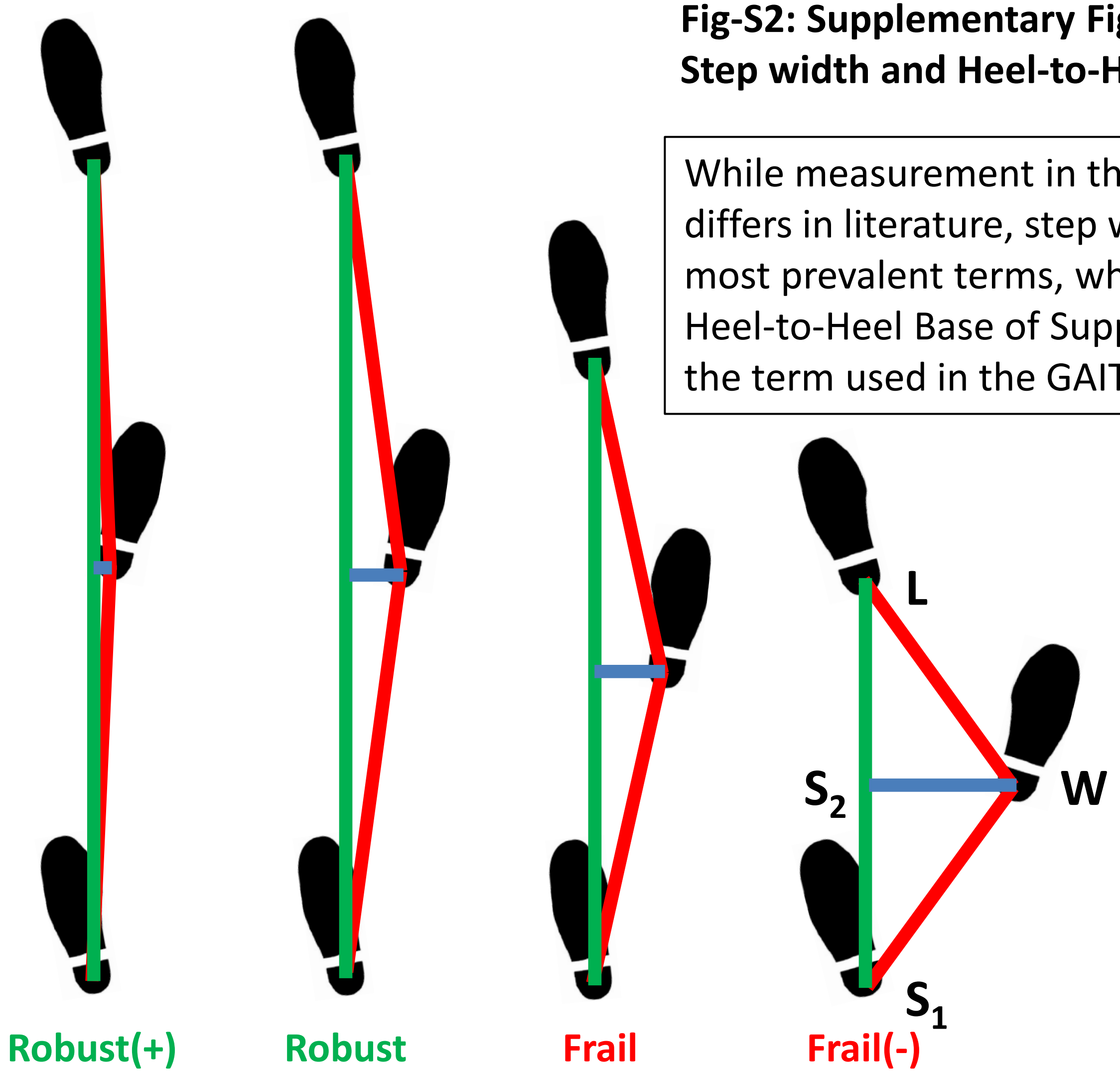

**S_1L**	**Stride length**
**S_2W**	**Step width**

Robust(+): Exemplary stride of a high performing Robust person
Robust : Average stride of the Robust group
Frail : Average stride of the Frail group
Frail(-) : Exemplary stride of a low performing Frail person
