# Supplementary material for: Reference values of gait characteristics in community-dwelling older persons with different physical functional levels
Source: BMC Geriatr. 2022 Aug 29;22:713. doi: 10.1186/s12877-022-03373-0 (PMC9422159; doi:10.1186/s12877-022-03373-0)

Fig-S3: Supplementary Figure 3:
Range of mean preferred gait speed between functional levels within a age group

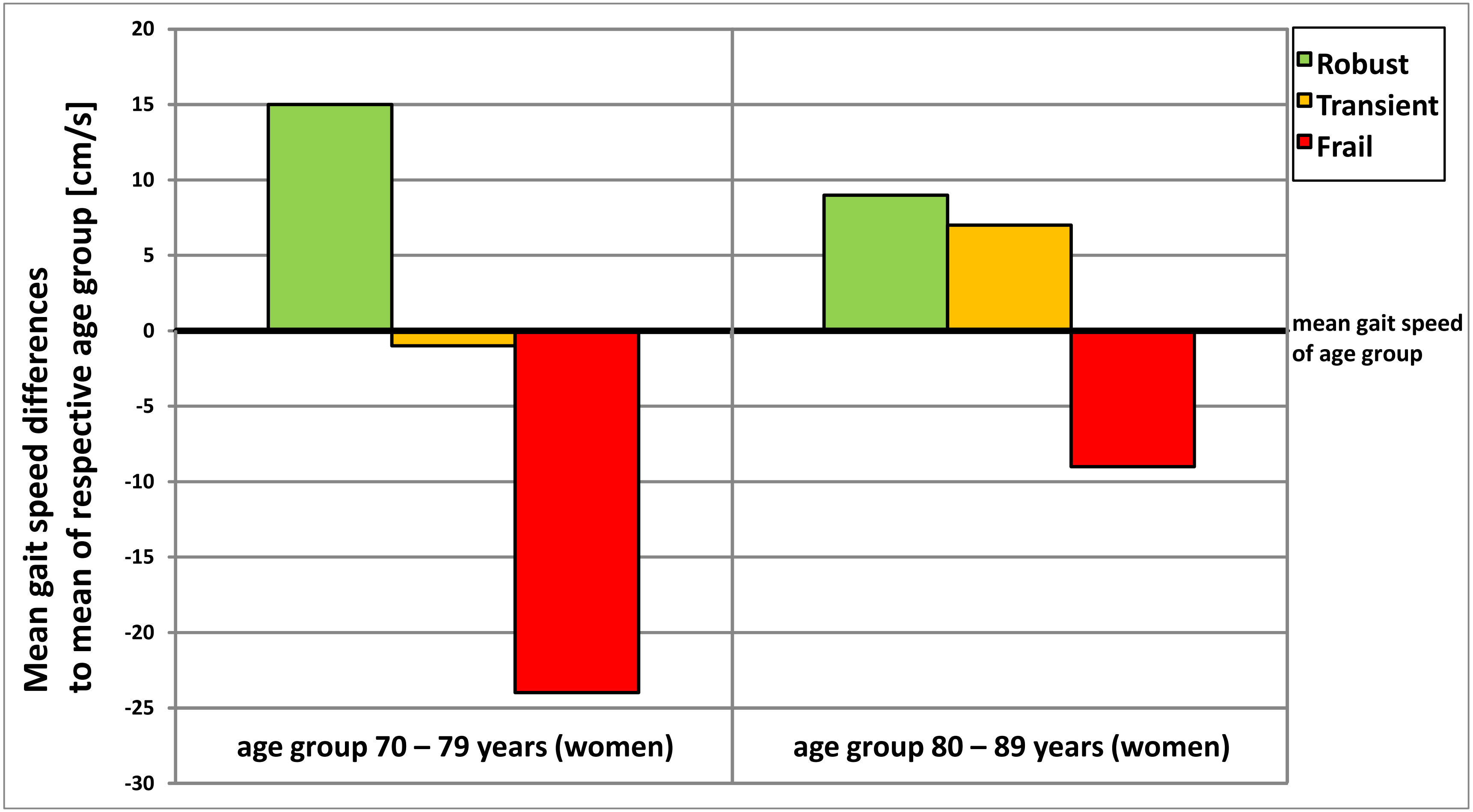

Supplement: Supplementary file 3 — Additional file 3: Supplementary Fig. 3. [file 12877_2022_3373_MOESM3_ESM.pdf]
