# Supplementary material for: Reference values of gait characteristics in community-dwelling older persons with different physical functional levels
Source: BMC Geriatr. 2022 Aug 29;22:713. doi: 10.1186/s12877-022-03373-0 (PMC9422159; doi:10.1186/s12877-022-03373-0)
Supplement: Supplementary file 4 — Additional file 4: Supplementary Table 1 to Supplementary Table 7. [file 12877_2022_3373_MOESM4_ESM.docx]

**Table S1: Supplementary Table 1a and Table 1b: Gait parameters at preferred speed by functional ability, sex and age group 70-79years (70-79y)**

| **Supplementary Table 1a: Gait parameters at preferred speed by age 70-79y, sex and Short Physical Performance Battery (SPPB) score** | | | | | | | | |
| --- | --- | --- | --- | --- | --- | --- | --- | --- |
| **Pref n=408** | **Male** | | | | **Female** | | | |
| **Functional ability** | **70-79y Total** | **70-79y Robust** | **70-79y Transient** | **70-79y Frail** | **70-79y Total** | **70-79y Robust** | **70-79y Transient** | **70-79y Frail** |
| **Gait Parameter** | n=149 | n=53 | n=68 | n=28 | n=259 | n=86 | n=121 | n=52 |
| 1. Velocity [cm/s] | 117.4 ± 24.6 | 133.6 ± 19.0 | 115.9 ± 19.2 | 90.8 ± 21.6 | 115.9 ± 22.5 | 131.3 ± 16.4 | 115.1 ± 18.1 | 92.3 ± 19.4 |
| 2. Stride length [cm] | 130.8 ± 21.9 | 144.1 ± 15.2 | 130.2 ± 17.3 | 107.2 ± 22.7 | 122.3 ± 16.9 | 132.3 ± 13.4 | 122.5 ± 13.4 | 105.3 ± 16.1 |
| 3. Walk ratio^1^ [cm/(steps/min)] | 0.61 ± 0.10 | 0.65 ± 0.07 | 0.61 ± 0.09 | 0.53 ± 0.11 | 0.54 ± 0.07 | 0.56 ± 0.06 | 0.55 ± 0.06 | 0.50 ± 0.08 |
| 4. Single support time [ms] | 408 ± 31 | 407 ± 27 | 408 ± 33 | 412 ± 35 | 390 ± 32 | 382 ± 24 | 392 ± 30 | 399 ± 45 |
| 5. Double support time [ms] | 312 ± 72 | 273 ± 46 | 317 ± 58 | 375 ± 94 | 291 ± 66 | 248 ± 38 | 291 ± 50 | 361 ± 75 |
| 6. Step width^2^ [cm] | 11.1 ± 2.9 | 10.6 ± 2.8 | 11.0 ± 2.6 | 12.5 ± 3.6 | 9.0 ± 3.2 | 7.8 ± 2.7 | 8.9 ± 2.7 | 11.4 ± 3.7 |
| 7. Step width^2^ CV^3^ [%] | 20.8 ± 12.8 | 20.1 ± 13.5 | 22.1 ± 12.7 | 19.1 ± 11.8 | 27.5 ± 16.5 | 30.0 ± 18.5 | 27.5 ± 14.9 | 23.5 ± 16.1 |
| 8. Stride length CV^3^ [%] | 3.8 ± 2.0 | 3.1 ± 1.3 | 3.7 ± 2.1 | 5.3 ± 2.1 | 3.6 ± 1.8 | 3.1 ± 1.6 | 3.5 ± 1.6 | 4.6 ± 2.2 |
|  | | | | | | | | |
| **Supplementary Table 1b: Gait parameters at preferred speed by age 70-79y, sex and LUCAS Functional Ability Index (LUCAS FAI)** | | | | | | | | |
| **Pref n=408** | **Male** | | | | **Female** | | | |
| **Functional ability** | **70-79y Total** | **70-79y Robust** | **70-79y Transient** | **70-79y Frail** | **70-79y Total** | **70-79y Robust** | **70-79y Transient** | **70-79y Frail** |
| **Gait Parameter** | n=149 | n=40 | n=65 | n=44 | n=259 | n=51 | n=138 | n=70 |
| 1. Velocity [cm/s] | 117.4 ± 24.6 | 138.3 ± 17.0 | 114.4 ± 21.8 | 103.0 ± 22.1 | 115.9 ± 22.5 | 134.1 ± 17.5 | 116.0 ± 19.9 | 102.4 ± 21.5 |
| 2. Stride length [cm] | 130.8 ± 21.9 | 147.9 ± 13.7 | 128.8 ± 19.9 | 118.2 ± 21.3 | 122.3 ± 16.9 | 135.0 ± 12.9 | 122.4 ± 15.0 | 133.0 ± 17.2 |
| 3. Walk ratio^1^ [cm/(steps/min)] | 0.61 ± 0.10 | 0.66 ± 0.07 | 0.61 ± 0.09 | 0.57 ± 0.11 | 0.54 ± 0.07 | 0.57 ± 0.06 | 0.54 ± 0.06 | 0.52 ± 0.07 |
| 4. Single support time [ms] | 408 ± 31 | 403 ± 25 | 411 ± 31 | 409 ± 37 | 390 ± 32 | 386 ± 25 | 388 ± 31 | 398 ± 39 |
| 5. Double support time [ms] | 312 ± 72 | 265 ± 43 | 318 ± 65 | 348 ± 80 | 291 ± 66 | 240 ± 43 | 291 ± 58 | 328 ± 70 |
| 6. Step width^2^ [cm] | 11.1 ± 2.9 | 10.9 ± 2.2 | 10.8 ± 3.3 | 11.9 ± 2.9 | 9.0 ± 3.2 | 8.3 ± 2.5 | 9.0 ± 3.0 | 9.5 ± 3.9 |
| 7. Step width^2^ CV^3^ [%] | 20.8 ± 12.8 | 20.7 ± 10.9 | 23.9 ± 14.6 | 16.4 ± 10.2 | 27.5 ± 16.5 | 28.8 ± 20.0 | 26.9 ± 14.7 | 27.7 ± 17.4 |
| 8. Stride length CV^3^ [%] | 3.8 ± 2.0 | 2.6 ± 1.2 | 4.3 ± 2.1 | 4.2 ± 1.9 | 3.6 ± 1.8 | 2.9 ± 1.6 | 3.6 ± 1.6 | 4.0 ± 2.1 |

R: ROBUST, T: TRANSIENT, F: FRAIL
^1^formula: (Stride Length / 2) / (Number of Steps/min)
^2^the GAITRite system uses the term “Heel to Heel Base of Support”
^3^coefficient of variance; formula: (Standard Deviation / Mean) * 100

**Table S1: Supplementary Table 1c and Table 1d: Gait parameters at preferred speed by functional ability, sex and age group 80years+ (80y+)**

| **Supplementary Table 1c: Gait parameters at preferred speed by age 80y+, sex and Short Physical Performance Battery (SPPB) score** | | | | | | | | |
| --- | --- | --- | --- | --- | --- | --- | --- | --- |
| **Pref n=234** | **Male** | | | | **Female** | | | |
| **Functional ability** | **80y+ Total** | **80y+ Robust** | **80y+ Transient** | **80y+ Frail** | **80y+ Total** | **80y+ Robust** | **80y+ Transient** | **80y+ Frail** |
| **Gait Parameter** | n=84 | n=14 | n=36 | n=34 | n=150 | n=21 | n=59 | n=70 |
| 1. Velocity [cm/s] | 102.2 ± 23.9 | 125.5 ± 18.7 | 108.2 ± 19.0 | 86.3 ± 19.6 | 99.7 ± 20.2 | 117.8 ± 16.9 | 107.2 ± 14.9 | 87.8 ± 17.8 |
| 2. Stride length [cm] | 117.0 ± 21.2 | 134.5 ± 16.9 | 123.9 ± 17.2 | 102.4 ± 17.3 | 108.4 ± 16.1 | 121.8 ± 12.9 | 114.3 ± 11.0 | 99.3 ± 15.6 |
| 3. Walk ratio^1^ [cm/(steps/min)] | 0.56 ± 0.09 | 0.60 ± 0.09 | 0.60 ± 0.09 | 0.51 ± 0.08 | 0.49 ± 0.07 | 0.53 ± 0.05 | 0.51 ± 0.06 | 0.47 ± 0.07 |
| 4. Single support time [ms] | 411 ± 36 | 400 ± 33 | 413 ± 35 | 412 ± 39 | 393 ± 31 | 390 ± 24 | 390 ± 30 | 395 ± 33 |
| 5. Double support time [ms] | 343 ± 74 | 278 ± 43 | 333 ± 71 | 380 ± 66 | 318 ± 68 | 263 ± 36 | 296 ± 47 | 353 ± 73 |
| 6. Step width^2^ [cm] | 11.9 ± 4.0 | 10.4 ± 1.8 | 10.7 ± 3.8 | 13.9 ± 4.1 | 9.6 ± 3.2 | 8.5 ± 2.2 | 9.3 ± 2.9 | 10.3 ± 3.6 |
| 7. Step width^2^ CV^3^ [%] | 22.4 ± 12.8 | 19.5 ± 7.4 | 25.1 ± 14.8 | 20.7 ± 12.2 | 27.2 ± 15.0 | 22.8 ± 8.6 | 28.9 ± 16.1 | 27.1 ± 15.4 |
| 8. Stride length CV^3^ [%] | 5.0 ± 2.8 | 3.8 ± 1.9 | 4.5 ± 2.1 | 6.0 ± 3.5 | 4.7 ± 2.8 | 3.4 ± 1.3 | 4.0 ± 1.8 | 5.7 ± 3.5 |
|  | | | | | | | | |
| **Supplementary Table 1d: Gait parameters at preferred speed by age 80y+, sex and LUCAS Functional Ability Index (LUCAS FAI)** | | | | | | | | |
| **Pref n=234** | **Male** | | | | **Female** | | | |
| **Functional ability** | **80y+ Total** | **80y+ Robust** | **80y+ Transient** | **80y+ Frail** | **80y+ Total** | **80y+ Robust** | **80y+ Transient** | **80y+ Frail** |
| **Gait Parameter** | n=84 | n=6 | n=50 | n=28 | n=150 | n=7 | n=70 | n=73 |
| 1. Velocity [cm/s] | 102.2 ± 23.9 | 131.4 ± 18.0 | 104.9 ± 23.1 | 91.2 ± 19.9 | 99.7 ± 20.2 | 109.8 ± 21.3 | 107.9 ± 18.6 | 90.8 ± 17.8 |
| 2. Stride length [cm] | 117.0 ± 21.2 | 143.2 ± 10.2 | 120.2 ± 19.1 | 105.7 ± 19.9 | 108.4 ± 16.1 | 114.1 ± 16.4 | 114.9 ± 14.7 | 101.5 ± 14.7 |
| 3. Walk ratio^1^ [cm/(steps/min)] | 0.56 ± 0.09 | 0.65 ± 0.04 | 0.58 ± 0.08 | 0.51 ± 0.10 | 0.49 ± 0.07 | 0.49 ± 0.05 | 0.51 ± 0.06 | 0.48 ± 0.07 |
| 4. Single support time [ms] | 411 ± 36 | 413 ± 30 | 415 ± 35 | 403 ± 40 | 393 ± 31 | 380 ± 22 | 392 ± 29 | 395 ± 33 |
| 5. Double support time [ms] | 343 ± 74 | 276 ± 51 | 338 ± 75 | 365 ± 65 | 318 ± 68 | 290 ± 51 | 295 ± 63 | 342 ± 67 |
| 6. Step width^2^ [cm] | 11.9 ± 4.0 | 9.3 ± 2.9 | 11.0 ± 3.2 | 14.3 ± 4.5 | 9.6 ± 3.2 | 9.3 ± 3.8 | 9.3 ± 3.0 | 9.9 ± 3.3 |
| 7. Step width^2^ CV^3^ [%] | 22.4 ± 12.8 | 21.7 ± 12.8 | 23.0 ± 12.1 | 21.4 ± 14.5 | 27.2 ± 15.0 | 24.2 ± 11.7 | 27.8 ± 14.6 | 26.9 ± 15.7 |
| 8. Stride length CV^3^ [%] | 5.0 ± 2.8 | 3.5 ± 1.8 | 4.3 ± 2.1 | 6.5 ± 3.5 | 4.7 ± 2.8 | 3.6 ± 1.0 | 4.2 ± 2.0 | 5.3 ± 3.5 |

R: ROBUST, T: TRANSIENT, F: FRAIL
^1^formula: (Stride Length / 2) / (Number of Steps/min)
^2^the GAITRite system uses the term “Heel to Heel Base of Support”
^3^coefficient of variance; formula: (Standard Deviation / Mean) * 100

**Table S2: Supplementary Table 2a and Table 2b: Gait parameters at preferred speed by functional ability (male/female)**

| **Supplementary Table 2a: Gait parameters at preferred speed by Short Physical Performance Battery (SPPB) score (male/female)** | | | | | | | | | | | | | | |
| --- | --- | --- | --- | --- | --- | --- | --- | --- | --- | --- | --- | --- | --- | --- |
| **Pref n=642** | **Male** | | | **Female** | | | **p-value Male** | | | | **p-value Female** | | | |
| **Functional ability** | **SPPB 12-11 Robust** | **SPPB 10-8 Transient** | **SPPB 7-0 Frail** | **SPPB 12-11 Robust** | **SPPB 10-8 Transient** | **SPPB 7-0 Frail** | **total** | **R/T** | **R/F** | **T/F** | **total** | **R/T** | **R/F** | **T/F** |
| **Gait Parameter** | n=67 | n=104 | n=62 | n=107 | n=180 | n=122 |  |  |  |  |  |  |  |  |
| 1. Velocity [cm/s] | 131.9 ± 19.1 | 113.2 ± 19.4 | 88.3 ± 20.5 | 128.7 ± 17.3 | 112.5 ± 17.5 | 89.7 ± 18.6 | **<0.001** | **<0.001** | **<0.001** | **<0.001** | **<0.001** | **<0.001** | **<0.001** | **<0.001** |
| 2. Stride length [cm] | 142.1 ± 15.9 | 128.0 ± 17.4 | 104.6 ± 19.9 | 130.2 ± 13.9 | 119.8 ± 13.2 | 101.8 ± 16.0 | **<0.001** | **<0.001** | **<0.001** | **<0.001** | **<0.001** | **<0.001** | **<0.001** | **<0.001** |
| 3. Walk ratio^1^ [cm/(steps/min)] | 0.64 ± 0.08 | 0.61 ± 0.09 | 0.52 ± 0.09 | 0.55 ± 0.06 | 0.53 ± 0.06 | 0.48 ± 0.08 | **<0.001** | **0.011** | **<0.001** | **<0.001** | **<0.001** | **0.027** | **<0.001** | **<0.001** |
| 4. Single support time [ms] | 405 ± 28 | 410 ± 34 | 412 ± 37 | 384 ± 24 | 391 ± 30 | 397 ± 39 | 0.458 | 0.321 | 0.226 | 0.714 | **0.006** | **0.015** | **0.002** | 0.181 |
| 5. Double support time [ms] | 274 ± 45 | 323 ± 63 | 377 ± 79 | 251 ± 38 | 293 ± 49 | 357 ± 74 | **<0.001** | **<0.001** | **<0.001** | **<0.001** | **<0.001** | **<0.001** | **<0.001** | **<0.001** |
| 6. Step width^2^ [cm] | 10.5 ± 2.6 | 10.9 ± 3.1 | 13.3 ± 3.9 | 7.9 ± 2.6 | 9.0 ± 2.7 | 10.7 ± 3.7 | **<0.001** | 0.467 | **<0.001** | **<0.001** | **<0.001** | **0.001** | **<0.001** | **<0.001** |
| 7. Step width^2^ CV^3^ [%] | 20.0 ± 12.4 | 23.1 ± 13.5 | 20.0 ± 11.9 | 28.6 ± 17.2 | 27.9 ± 15.3 | 25.5 ±15.7 | 0.179 | 0.130 | 0.986 | 0.130 | 0.299 | 0.754 | 0.168 | 0.187 |
| 8. Stride length CV^3^ [%] | 3.2 ± 1.5 | 4.0 ± 2.1 | 5.7 ± 2.9 | 3.1 ± 1.6 | 3.7 ±1.6 | 5.2 ± 3.1 | **<0.001** | **0.005** | **<0.001** | **<0.001** | **<0.001** | **0.006** | **<0.001** | **<0.001** |
|  | | | | | | | | | | | | | | |
| **Supplementary Table 2b: Gait parameters at preferred speed by LUCAS Functional Ability Index (LUCAS FAI) (male/female)** | | | | | | | | | | | | | | |
| **Pref n=642** | **Male** | | | **Female** | | | **p-value Male** | | | | **p-value Female** | | | |
| **Functional ability** | **LUCAS FAI Robust** | **LUCAS FAI Transient** | **LUCAS FAI Frail** | **LUCAS FAI Robust** | **LUCAS FAI Transient** | **LUCAS FAI Frail** | **total** | **R/T** | **R/F** | **T/F** | **total** | **R/T** | **R/F** | **T/F** |
| **Gait Parameter** | n=46 | n=115 | n=72 | n=58 | n=208 | n=143 |  |  |  |  |  |  |  |  |
| 1. Velocity [cm/s] | 137.4 ± 17.1 | 110.3 ± 22.8 | 98.4 ± 21.9 | 131.2 ± 19.5 | 113.3 ± 19.8 | 96.5 ± 20.5 | **<0.001** | **<0.001** | **<0.001** | **0.001** | **<0.001** | **<0.001** | **<0.001** | **<0.001** |
| 2. Stride length [cm] | 147.3 ± 13.3 | 125.1 ± 19.9 | 113.4 ± 21.5 | 132.4 ± 14.9 | 119.8 ± 15.3 | 107.1 ± 16.9 | **<0.001** | **<0.001** | **<0.001** | **<0.001** | **<0.001** | **<0.001** | **<0.001** | **<0.001** |
| 3. Walk ratio^1^ [cm/(steps/min)] | 0.66 ± 0.06 | 0.60 ± 0.09 | 0.55 ± 0.11 | 0.56 ± 0.06 | 0.53 ± 0.06 | 0.50 ± 0.07 | **<0.001** | **<0.001** | **<0.001** | **0.001** | **<0.001** | **0.004** | **<0.001** | **<0.001** |
| 4. Single support time [ms] | 405 ± 26 | 413 ± 33 | 407 ± 38 | 386 ± 25 | 389 ± 30 | 396 ± 36 | 0.284 | 0.145 | 0.760 | 0.243 | **0.039** | 0.456 | **0.018** | **0.045** |
| 5. Double support time [ms] | 267 ± 44 | 326 ± 70 | 354 ± 75 | 246 ± 47 | 292 ± 60 | 335 ± 68 | **<0.001** | **<0.001** | **<0.001** | **0.011** | **<0.001** | **<0.001** | **<0.001** | **<0.001** |
| 6. Step width^2^ [cm] | 10.7 ± 2.3 | 10.8 ± 3.2 | 12.8 ± 3.7 | 8.4 ± 2.7 | 9.1 ± 3.0 | 9.7 ± 3.6 | **<0.001** | 0.696 | **<0.001** | **<0.001** | **0.025** | 0.110 | **0.006** | 0.100 |
| 7. Step width^2^ CV^3^ [%] | 20.8 ± 11.0 | 23.5 ± 13.5 | 18.4 ± 12.2 | 28.2 ± 19.1 | 27.2 ± 14.6 | 27.3 ± 16.5 | **0.027** | 0.231 | 0.279 | **0.010** | 0.908 | 0.663 | 0.726 | 0.965 |
| 8. Stride length CV^3^ [%] | 2.7 ± 1.3 | 4.3 ± 2.1 | 5.1 ± 2.9 | 3.0 ± 1.6 | 3.8 ± 1.7 | 4.6 ± 3.0 | **<0.001** | **<0.001** | **<0.001** | **0.028** | **<0.001** | **0.003** | **<0.001** | **0.002** |

R: ROBUST, T: TRANSIENT, F: FRAIL
^1^formula: (Stride Length / 2) / (Number of Steps/min)
^2^the GAITRite system uses the term “Heel to Heel Base of Support”
^3^coefficient of variance; formula: (Standard Deviation / Mean) * 100

**Table S3: Supplementary Table 3a and Table 3b: Gait parameters at fast speed by functional ability (male/female)**

| **Supplementary Table 3a: Gait parameters at fast speed by Short Physical Performance Battery (SPPB) score (male/female)** | | | | | | | | | | | | | | |
| --- | --- | --- | --- | --- | --- | --- | --- | --- | --- | --- | --- | --- | --- | --- |
| **Fast n=601** | **Male** | | | **Female** | | | **p-value Male** | | | | **p-value Female** | | | |
| **Functional ability** | **SPPB 12-11 Robust** | **SPPB 10-8 Transient** | **SPPB 7-0 Frail** | **SPPB 12-11 Robust** | **SPPB 10-8 Transient** | **SPPB 7-0 Frail** | **total** | **R/T** | **R/F** | **T/F** | **total** | **R/T** | **R/F** | **T/F** |
| **Gait Parameter** | n=65 | n=100 | n=56 | n=103 | n=172 | n=105 |  |  |  |  |  |  |  |  |
| 1. Velocity [cm/s] | 177.8 ± 24.0 | 155.5 ± 25.7 | 125.1 ± 23.5 | 169.0 ± 20.2 | 150.8 ± 21.6 | 123.1 ± 21.6 | **<0.001** | **<0.001** | **<0.001** | **<0.001** | **<0.001** | **<0.001** | **<0.001** | **<0.001** |
| 2. Stride length [cm] | 163.6 ± 16.9 | 149.6 ± 17.2 | 128.1 ± 20.2 | 145.0 ± 14.5 | 134.3 ± 15.0 | 118.2 ± 16.2 | **<0.001** | **<0.001** | **<0.001** | **<0.001** | **<0.001** | **<0.001** | **<0.001** | **<0.001** |
| 3. Walk ratio^1^ [cm/(steps/min)] | 0.63 ± 0.08 | 0.60 ± 0.08 | 0.55 ± 0.09 | 0.52 ± 0.07 | 0.50 ± 0.07 | 0.48 ± 0.07 | **<0.001** | **0.050** | **<0.001** | **<0.001** | **<0.001** | **0.026** | **<0.001** | **0.004** |
| 4. Single support time [ms] | 365 ± 27 | 371 ± 31 | 379 ± 30 | 343 ± 26 | 346 ± 28 | 357 ± 32 | **0.031** | 0.175 | **0.006** | 0.124 | **0.001** | 0.397 | **0.001** | **0.003** |
| 5. Double support time [ms] | 193 ± 38 | 231 ± 55 | 275 ± 61 | 175 ± 34 | 207 ± 42 | 257 ± 56 | **<0.001** | **<0.001** | **<0.001** | **<0.001** | **<0.001** | **<0.001** | **<0.001** | **<0.001** |
| 6. Step width^2^ [cm] | 10.3 ± 2.8 | 10.2 ± 2.9 | 11.9 ± 3.9 | 8.1 ± 2.4 | 8.6 ± 2.8 | 10.0 ± 3.4 | **0.004** | 0.776 | **0.013** | **0.005** | **<0.001** | 0.166 | **<0.001** | **0.001** |
| 7. Step width^2^ CV^3^ [%] | 22.4 ± 15.6 | 25.0 ± 17.7 | 23.5 ± 12.8 | 27.8 ± 13.8 | 28.8 ± 17.5 | 25.5 ±14.6 | 0.568 | 0.326 | 0.677 | 0.563 | 0.239 | 0.606 | 0.252 | 0.106 |
| 8. Stride length CV^3^ [%] | 2.8 ± 1.9 | 3.7 ± 1.7 | 4.5 ± 2.5 | 2.8 ± 1.6 | 3.5 ± 1.8 | 4.1 ± 1.8 | **<0.001** | **0.002** | **<0.001** | **0.030** | **<0.001** | **0.001** | **<0.001** | **0.019** |
|  | | | | | | | | | | | | | | |
| **Supplementary Table 3b: Gait parameters at fast speed by LUCAS Functional Ability Index (LUCAS FAI) (male/female)** | | | | | | | | | | | | | | |
| **Fast n=601** | **Male** | | | **Female** | | | **p-value Male** | | | | **p-value Female** | | | |
| **Functional ability** | **LUCAS FAI Robust** | **LUCAS FAI Transient** | **LUCAS FAI Frail** | **LUCAS FAI Robust** | **LUCAS FAI Transient** | **LUCAS FAI Frail** | **total** | **R/T** | **R/F** | **T/F** | **total** | **R/T** | **R/F** | **T/F** |
| **Gait Parameter** | n=46 | n=110 | n=65 | n=58 | n=196 | n=126 |  |  |  |  |  |  |  |  |
| 1. Velocity [cm/s] | 184.4 ± 22.7 | 152.6 ± 28.2 | 136.2 ± 25.8 | 170.4 ± 22.4 | 150.8 ± 23.7 | 133.5 ± 26.1 | **<0.001** | **<0.001** | **<0.001** | **<0.001** | **<0.001** | **<0.001** | **<0.001** | **<0.001** |
| 2. Stride length [cm] | 167.7 ± 14.2 | 147.2 ± 19.2 | 136.5 ± 22.5 | 146.3 ± 15.1 | 134.3 ± 16.4 | 124.2 ± 17.7 | **<0.001** | **<0.001** | **<0.001** | **0.001** | **<0.001** | **<0.001** | **<0.001** | **<0.001** |
| 3. Walk ratio^1^ [cm/(steps/min)] | 0.64 ± 0.07 | 0.60 ± 0.08 | 0.57 ± 0.10 | 0.52 ± 0.06 | 0.50 ± 0.07 | 0.49 ± 0.07 | **0.001** | **0.002** | **<0.001** | 0.135 | **0.002** | **0.030** | **<0.001** | 0.053 |
| 4. Single support time [ms] | 364 ± 27 | 372 ± 30 | 376 ± 31 | 344 ± 25 | 347 ± 29 | 353 ± 32 | 0.113 | 0.141 | **0.035** | 0.360 | 0.076 | 0.603 | 0.065 | 0.055 |
| 5. Double support time [ms] | 185 ± 43 | 234 ± 57 | 259 ± 57 | 173 ± 39 | 207 ± 48 | 238 ± 55 | **<0.001** | **<0.001** | **<0.001** | **0.005** | **<0.001** | **<0.001** | **<0.001** | **<0.001** |
| 6. Step width^2^ [cm] | 10.2 ± 2.6 | 10.4 ± 3.0 | 11.5 ± 3.8 | 8.4 ± 2.5 | 8.8 ± 2.9 | 9.1 ± 3.3 | 0.054 | 0.662 | **0.036** | **0.043** | 0.386 | 0.344 | 0.142 | 0.472 |
| 7. Step width^2^ CV^3^ [%] | 19.9 ± 9.9 | 26.1 ± 17.7 | 22.9 ± 15.8 | 25.2 ± 13.1 | 28.2 ± 15.3 | 27.8 ± 17.6 | 0.069 | **0.006** | 0.263 | 0.222 | 0.449 | 0.181 | 0.330 | 0.807 |
| 8. Stride length CV^3^ [%] | 2.5 ± 1.4 | 3.7 ± 1.8 | 4.5 ± 2.4 | 2.7 ± 1.6 | 3.5 ± 1.8 | 3.8 ± 1.8 | **<0.001** | **<0.001** | **<0.001** | **0.024** | **0.001** | **0.003** | **<0.001** | 0.167 |

R: ROBUST, T: TRANSIENT, F: FRAIL
^1^formula: (Stride Length / 2) / (Number of Steps/min)
^2^the GAITRite system uses the term “Heel to Heel Base of Support”
^3^coefficient of variance; formula: (Standard Deviation / Mean) * 100

**Table S4: Supplementary Table 4a and Table 4b: Gait parameters at preferred speed by functional ability (70-79ys/80-94ys)**

| **Supplementary Table 4a: Gait parameters at preferred speed by Short Physical Performance Battery (SPPB) score (70-79ys/80-94ys)** | | | | | | | | | | | | | | |
| --- | --- | --- | --- | --- | --- | --- | --- | --- | --- | --- | --- | --- | --- | --- |
| **Pref n=642** | **70-79ys** | | | **80-94ys** | | | **p-value 70-79ys** | | | | **p-value 80-94ys** | | | |
| **Functional ability** | **SPPB 12-11 Robust** | **SPPB 10-8 Transient** | **SPPB 7-0 Frail** | **SPPB 12-11 Robust** | **SPPB 10-8 Transient** | **SPPB 7-0 Frail** | **total** | **R/T** | **R/F** | **T/F** | **total** | **R/T** | **R/F** | **T/F** |
| **Gait Parameter** | n=139 | n=189 | n=80 | n=35 | n=95 | n=104 |  |  |  |  |  |  |  |  |
| 1. Velocity [cm/s] | 132.2 ± 17.4 | 115.4 ± 18.4 | 91.7 ± 20.1 | 120.9 ± 17.8 | 107.6 ± 16.5 | 87.3 ± 18.3 | **<0.001** | **<0.001** | **<0.001** | **<0.001** | **<0.001** | **<0.001** | **<0.001** | **<0.001** |
| 2. Stride length [cm] | 136.8 ± 15.2 | 125.3 ± 15.3 | 106.0 ± 18.5 | 126.9 ± 15.7 | 118.0 ± 14.4 | 100.3 ± 16.1 | **<0.001** | **<0.001** | **<0.001** | **<0.001** | **<0.001** | **0.003** | **<0.001** | **<0.001** |
| 3. Walk ratio^1^ [cm/(steps/min)] | 0.59 ± 0.08 | 0.57 ± 0.08 | 0.51 ± 0.09 | 0.56 ± 0.08 | 0.54 ± 0.08 | 0.48 ± 0.08 | **<0.001** | **0.012** | **<0.001** | **<0.001** | **<0.001** | 0.346 | **<0.001** | **<0.001** |
| 4. Single support time [ms] | 391 ± 28 | 398 ± 32 | 404 ± 42 | 394 ± 28 | 399 ± 34 | 401 ± 36 | **0.027** | 0.056 | **0.022** | 0.281 | 0.564 | 0.419 | 0.286 | 0.704 |
| 5. Double support time [ms] | 258 ± 43 | 301 ± 54 | 366 ± 82 | 269 ± 39 | 310 ± 60 | 362 ± 71 | **<0.001** | **<0.001** | **<0.001** | **<0.001** | **<0.001** | **<0.001** | **<0.001** | **<0.001** |
| 6. Step width^2^ [cm] | 8.9 ± 3.1 | 9.6 ± 2.8 | 11.8 ± 3.7 | 9.2 ± 2.2 | 9.8 ± 3.3 | 11.5 ± 4.1 | **<0.001** | **0.016** | **<0.001** | **<0.001** | **0.001** | 0.250 | **<0.001** | **0.002** |
| 7. Step width^2^ CV^3^ [%] | 26.2 ± 17.4 | 25.5 ± 14.4 | 21.9 ± 14.8 | 21.5 ± 8.2 | 27.4 ± 15.6 | 25.0 ± 14.6 | 0.125 | 0.702 | 0.066 | 0.063 | 0.101 | **0.006** | 0.080 | 0.258 |
| 8. Stride length CV^3^ [%] | 3.1 ± 1.5 | 3.6 ± 1.8 | 4.8 ± 2.2 | 3.6 ± 1.6 | 4.2 ± 1.9 | 5.8 ± 3.5 | **<0.001** | **0.003** | **<0.001** | **<0.001** | **<0.001** | 0.111 | **<0.001** | **<0.001** |
|  | | | | | | | | | | | | | | |
| **Supplementary Table 4b: Gait parameters at preferred speed by LUCAS Functional Ability Index (LUCAS FAI) (70-79ys/80-94ys)** | | | | | | | | | | | | | | |
| **Pref n=642** | **70-79ys** | | | **80-94ys** | | | **p-value 70-79ys** | | | | **p-value 80-94ys** | | | |
| **Functional ability** | **LUCAS FAI Robust** | **LUCAS FAI Transient** | **LUCAS FAI Frail** | **LUCAS FAI Robust** | **LUCAS FAI Transient** | **LUCAS FAI Frail** | **total** | **R/T** | **R/F** | **T/F** | **total** | **R/T** | **R/F** | **T/F** |
| **Gait Parameter** | n=91 | n=203 | n=114 | n=13 | n=120 | n=101 |  |  |  |  |  |  |  |  |
| 1. Velocity [cm/s] | 136.0 ± 17.3 | 115.5 ± 20.5 | 102.7 ± 21.7 | 119.8 ± 22.1 | 106.6 ± 20.6 | 90.9 ± 18.3 | **<0.001** | **<0.001** | **<0.001** | **<0.001** | **<0.001** | **0.032** | **<0.001** | **<0.001** |
| 2. Stride length [cm] | 140.6 ± 14.7 | 124.4 ± 16.9 | 115.0 ± 18.9 | 127.5 ± 20.2 | 117.1 ± 16.8 | 102.7 ± 16.3 | **<0.001** | **<0.001** | **<0.001** | **<0.001** | **<0.001** | **0.040** | **<0.001** | **<0.001** |
| 3. Walk ratio^1^ [cm/(steps/min)] | 0.61 ± 0.08 | 0.56 ± 0.08 | 0.54 ± 0.09 | 0.57 ± 0.09 | 0.54 ± 0.08 | 0.49 ± 0.08 | **<0.001** | **<0.001** | **<0.001** | **0.035** | **<0.001** | 0.242 | **0.001** | **<0.001** |
| 4. Single support time [ms] | 394 ± 26 | 395 ± 33 | 402 ± 38 | 395 ± 30 | 401 ± 33 | 397 ± 35 | 0.146 | 0.776 | 0.082 | 0.097 | 0.634 | 0.528 | 0.832 | 0.401 |
| 5. Double support time [ms] | 251 ± 45 | 300 ± 62 | 336 ± 74 | 283 ± 49 | 313 ± 71 | 349 ± 67 | **<0.001** | **<0.001** | **<0.001** | **<0.001** | **<0.001** | 0.145 | **0.001** | **<0.001** |
| 6. Step width^2^ [cm] | 9.4 ± 2.7 | 9.6 ± 3.2 | 10.4 ± 3.7 | 9.3 ± 3.2 | 10.0 ± 3.2 | 11.1 ± 4.1 | **0.041** | 0.697 | **0.026** | **0.032** | **0.038** | 0.456 | 0.130 | **0.027** |
| 7. Step width^2^ CV^3^ [%] | 25.2 ± 17.0 | 26.0 ± 14.7 | 23.3 ± 15.9 | 23.0 ± 11.7 | 25.8 ± 13.8 | 25.4 ± 15.5 | 0.357 | 0.702 | 0.420 | 0.142 | 0.809 | 0.493 | 0.599 | 0.845 |
| 8. Stride length CV^3^ [%] | 2.8 ± 1.5 | 3.8 ± 1.8 | 4.1 ± 2.0 | 3.6 ± 1.4 | 4.3 ± 2.0 | 5.6 ± 3.5 | **<0.001** | **<0.001** | **<0.001** | 0.232 | **<0.001** | 0.234 | **0.041** | **0.001** |

R: ROBUST, T: TRANSIENT, F: FRAIL
^1^formula: (Stride Length / 2) / (Number of Steps/min)
^2^the GAITRite system uses the term “Heel to Heel Base of Support”
^3^coefficient of variance; formula: (Standard Deviation / Mean) * 100

**Table S5: Supplementary Table 5a and Table 5b: Gait parameters at fast speed by functional ability (70-79ys/80-94ys)**

| **Supplementary Table 5a: Gait parameters at fast speed by Short Physical Performance Battery (SPPB) score (70-79ys/80-94ys)** | | | | | | | | | | | | | | |
| --- | --- | --- | --- | --- | --- | --- | --- | --- | --- | --- | --- | --- | --- | --- |
| **Fast n=601** | **70-79ys** | | | **80-94ys** | | | **p-value 70-79ys** | | | | **p-value 80-94ys** | | | |
| **Functional ability** | **SPPB 12-11**  **Robust** | **SPPB 10-8**  **Transient** | **SPPB 7-0**  **Frail** | **SPPB 12-11**  **Robust** | **SPPB 10-8**  **Transient** | **SPPB 7-0**  **Frail** | **total** | **R/T** | **R/F** | **T/F** | **total** | **R/T** | **R/F** | **T/F** |
| **Gait Parameter** | n=134 | n=181 | n=72 | n=34 | n=91 | n=89 |  |  |  |  |  |  |  |  |
| 1. Velocity [cm/s] | 175.4 ± 20.6 | 155.4 ± 24.6 | 129.8 ± 20.6 | 160.7 ± 24.2 | 146.8 ± 19.2 | 119.0 ± 22.4 | **<0.001** | **<0.001** | **<0.001** | **<0.001** | **<0.001** | **0.001** | **<0.001** | **<0.001** |
| 2. Stride length [cm] | 154.6 ± 17.0 | 142.4 ± 17.4 | 126.0 ± 18.5 | 142.8 ± 18.5 | 135.1 ± 16.5 | 118.1 ± 17.4 | **<0.001** | **<0.001** | **<0.001** | **<0.001** | **<0.001** | **0.028** | **<0.001** | **<0.001** |
| 3. Walk ratio^1^ [cm/(steps/min)] | 0.57 ± 0.08 | 0.55 ± 0.09 | 0.51 ± 0.09 | 0.53 ± 0.10 | 0.52 ± 0.09 | 0.49 ± 0.08 | **<0.001** | **0.021** | **<0.001** | **0.006** | **0.023** | 0.578 | **0.019** | **0.020** |
| 4. Single support time [ms] | 351 ± 26 | 356 ± 31 | 361 ± 33 | 352 ± 36 | 354 ± 33 | 368 ± 33 | 0.099 | 0.186 | **0.040** | 0.272 | **0.007** | 0.740 | **0.017** | **0.005** |
| 5. Double support time [ms] | 180 ± 37 | 214 ± 50 | 256 ± 57 | 191 ± 36 | 219 ± 46 | 269 ± 58 | **<0.001** | **<0.001** | **<0.001** | **<0.001** | **<0.001** | **0.002** | **<0.001** | **<0.001** |
| 6. Step width^2^ [cm] | 8.9 ± 2.8 | 9.2 ± 3.0 | 10.6 ± 3.5 | 9.3 ± 2.6 | 9.1 ± 2.9 | 10.6 ± 3.9 | **<0.001** | 0.311 | **<0.001** | **0.002** | **0.004** | 0.709 | **0.026** | **0.002** |
| 7. Step width^2^ CV^3^ [%] | 25.5 ± 14.1 | 26.4 ± 17.3 | 24.4 ± 12.1 | 26.3 ± 16.9 | 29.5 ± 18.2 | 25.1 ± 15.4 | 0.657 | 0.645 | 0.582 | 0.316 | 0.203 | 0.372 | 0.703 | 0.080 |
| 8. Stride length CV^3^ [%] | 2.7 ± 1.5 | 3.3 ± 1.6 | 3.5 ± 1.5 | 3.4 ± 2.4 | 4.2 ± 2.0 | 4.8 ± 2.2 | **<0.001** | **0.001** | **<0.001** | 0.449 | **0.002** | **0.047** | **0.001** | **0.044** |
| **Supplementary Table 5b: Gait parameters at fast speed by LUCAS Functional Ability Index (LUCAS FAI) (70-79ys/80-94ys)** | | | | | | | | | | | | | | |
| **Fast n=601** | **70-79ys** | | | **80-94ys** | | | **p-value 70-79ys** | | | | **p-value 80-94ys** | | | |
| **Functional ability** | **Robust** | **Transient** | **Frail** | **Robust** | **Transient** | **Frail** | **total** | **R/T** | **R/F** | **T/F** | **total** | **R/T** | **R/F** | **T/F** |
| **Gait Parameter** | n=91 | n=190 | n=106 | n=13 | n=116 | n=85 |  |  |  |  |  |  |  |  |
| 1. Velocity [cm/s] | 179.3 ± 22.4 | 156.1 ± 24.0 | 141.4 ± 25.5 | 157.5 ± 22.6 | 143.8 ± 25.9 | 125.7 ± 24.0 | **<0.001** | **<0.001** | **<0.001** | **<0.001** | **<0.001** | 0.070 | **<0.001** | **<0.001** |
| 2. Stride length [cm] | 157.6 ± 17.0 | 142.1 ± 17.9 | 134.1 ± 19.9 | 142.7 ± 20.8 | 133.6 ± 18.3 | 121.3 ± 18.6 | **<0.001** | **<0.001** | **<0.001** | **<0.001** | **<0.001** | 0.095 | **<0.001** | **<0.001** |
| 3. Walk ratio^1^ [cm/(steps/min)] | 0.58 ± 0.08 | 0.54 ± 0.09 | 0.53 ± 0.09 | 0.54 ± 0.11 | 0.52 ± 0.09 | 0.49 ± 0.08 | **0.001** | **0.001** | **0.001** | 0.413 | **0.025** | 0.459 | 0.059 | **0.015** |
| 4. Single support time [ms] | 353 ± 25 | 354 ± 30 | 360 ± 33 | 353 ± 40 | 359 ± 34 | 363 ± 33 | 0.209 | 0.878 | 0.127 | 0.123 | 0.519 | 0.550 | 0.321 | 0.406 |
| 5. Double support time [ms] | 175 ± 41 | 211 ± 48 | 239 ± 58 | 201 ± 36 | 226 ± 60 | 254 ± 54 | **<0.001** | **<0.001** | **<0.001** | **<0.001** | **<0.001** | 0.144 | **0.001** | **0.001** |
| 6. Step width^2^ [cm] | 9.2 ± 2.6 | 9.2 ± 3.0 | 9.8 ± 3.4 | 9.0 ± 3.0 | 9.6 ± 3.0 | 10.0 ± 3.9 | 0.327 | 0.970 | 0.220 | 0.180 | 0.488 | 0.463 | 0.351 | 0.400 |
| 7. Step width^2^ CV^3^ [%] | 22.7 ± 12.3 | 28.0 ± 17.0 | 24.2 ± 14.2 | 24.0 ± 10.9 | 26.6 ± 14.9 | 28.5 ± 20.0 | **0.012** | **0.003** | 0.438 | 0.051 | 0.577 | 0.542 | 0.432 | 0.464 |
| 8. Stride length CV^3^ [%] | 2.5 ± 1.4 | 3.3 ± 1.6 | 3.4 ± 1.5 | 3.6 ± 1.9 | 4.1 ± 2.1 | 4.8 ± 2.3 | **<0.001** | **<0.001** | **<0.001** | 0.484 | **0.029** | 0.468 | 0.084 | **0.018** |

R: ROBUST, T: TRANSIENT, F: FRAIL
^1^formula: (Stride Length / 2) / (Number of Steps/min)
^2^the GAITRite system uses the term “Heel to Heel Base of Support”
^3^coefficient of variance; formula: (Standard Deviation / Mean) * 100

**Table S6: Supplementary Table 6a and Table 6b: Gait parameters at fast speed by functional ability, sex and age group 70-79y**

| **Supplementary Table 6a: Gait parameters at fast speed by age 70-79y, sex and Short Physical Performance Battery (SPPB) score** | | | | | | | | |
| --- | --- | --- | --- | --- | --- | --- | --- | --- |
| **Fast n=387** | **Male** | | | | **Female** | | | |
| **Functional ability** | **70-79y Total** | **70-79y Robust** | **70-79y Transient** | **70-79y Frail** | **70-79y Total** | **70-79y Robust** | **70-79y Transient** | **70-79y Frail** |
| **Gait Parameter** | n=142 | n=52 | n=65 | n=25 | n=245 | n=82 | n=116 | n=47 |
| 1. Velocity [cm/s] | 162.2 ± 30.0 | 181.5 ± 21.5 | 158.2 ± 28.1 | 132.4 ± 20.5 | 154.8 ± 25.8 | 171.5 ± 19.0 | 153.7 ± 22.3 | 128.4 ± 20.8 |
| 2. Stride length [cm] | 153.9 ± 21.0 | 166.4 ± 14.8 | 151.5 ± 18.4 | 133.9 ± 21.1 | 137.6 ± 17.0 | 147.1 ± 13.8 | 137.2 ± 14.6 | 121.8 ± 15.6 |
| 3. Walk ratio^1^ [cm/(steps/min)] | 0.61 ± 0.08 | 0.64 ± 0.07 | 0.61 ± 0.08 | 0.57 ± 0.11 | 0.51 ± 0.07 | 0.53 ± 0.07 | 0.51 ± 0.07 | 0.48 ± 0.07 |
| 4. Single support time [ms] | 369 ± 29 | 364 ± 23 | 370 ± 31 | 375 ± 30 | 347 ± 28 | 344 ± 25 | 348 ± 28 | 353 ± 31 |
| 5. Double support time [ms] | 221 ± 57 | 190 ± 37 | 229 ± 56 | 262 ± 59 | 204 ± 52 | 173 ± 35 | 206 ± 44 | 253 ± 56 |
| 6. Step width^2^ [cm] | 10.5 ± 3.0 | 10.4 ± 2.8 | 10.5 ± 3 | 10.7 ± 3.5 | 8.7 ± 2.9 | 7.9 ± 2.4 | 8.6 ± 2.7 | 10.6 ± 3.5 |
| 7. Step width^2^ CV^3^ [%] | 22.9 ± 15.3 | 21.3 ± 12.5 | 23.4 ± 17.9 | 25.3 ± 13.1 | 27.3 ± 15.2 | 28.2 ± 14.5 | 28.0 ± 16.9 | 24.0 ± 11.6 |
| 8. Stride length CV^3^ [%] | 3.1 ± 1.5 | 2.6 ± 1.4 | 3.2 ± 1.4 | 3.8 ± 1.8 | 3.1 ± 1.6 | 2.8 ± 1.5 | 3.3 ± 1.7 | 3.3 ± 1.4 |
|  | | | | | | | | |
| **Supplementary Table 6b: Gait parameters at fast speed by age 70-79y, sex and LUCAS Functional Ability Index (LUCAS FAI)** | | | | | | | | |
| **Fast n=387** | **Male** | | | | **Female** | | | |
| **Functional ability** | **70-79y Total** | **70-79y Robust** | **70-79y Transient** | **70-79y Frail** | **70-79y Total** | **70-79y Robust** | **70-79y Transient** | **70-79y Frail** |
| **Gait Parameter** | n=142 | n=40 | n=61 | n=41 | n=245 | n=51 | n=129 | n=65 |
| 1. Velocity [cm/s] | 162.2 ± 30.0 | 186.8 ± 23.1 | 159.5 ± 26.5 | 142.3 ± 23.8 | 154.8 ± 25.8 | 173.5 ± 20.2 | 154.5 ± 22.7 | 140.8 ± 26.6 |
| 2. Stride length [cm] | 153.9 ± 21.0 | 168.9 ± 14.3 | 152.6 ± 18.2 | 141.0 ± 21.3 | 137.6 ± 17.0 | 148.7 ± 13.3 | 137.2 ± 15.6 | 129.7 ± 17.8 |
| 3. Walk ratio^1^ [cm/(steps/min)] | 0.61 ± 0.08 | 0.64 ± 0.07 | 0.61 ± 0.07 | 0.58 ± 0.10 | 0.51 ± 0.07 | 0.53 ± 0.06 | 0.51 ± 0.07 | 0.50 ± 0.07 |
| 4. Single support time [ms] | 369 ± 29 | 363 ± 25 | 370 ± 28 | 373 ± 32 | 347 ± 28 | 346 ± 24 | 346 ± 27 | 351 ± 32 |
| 5. Double support time [ms] | 221 ± 57 | 183 ± 45 | 226 ± 52 | 250 ± 54 | 204 ± 52 | 168 ± 36 | 204 ± 44 | 232 ± 60 |
| 6. Step width^2^ [cm] | 10.5 ± 3.0 | 10.4 ± 2.6 | 10.5 ± 3.3 | 10.7 ± 3.0 | 8.7 ± 2.9 | 8.3 ± 2.3 | 8.7 ± 2.7 | 9.2 ± 3.6 |
| 7. Step width^2^ CV^3^ [%] | 22.9 ± 15.3 | 19.5 ± 10.2 | 26.9 ± 19.0 | 20.4 ± 11.5 | 27.3 ± 15.2 | 25.3 ± 13.3 | 28.5 ± 16.0 | 26.6 ± 15.2 |
| 8. Stride length CV^3^ [%] | 3.1 ± 1.5 | 2.3 ± 1.3 | 3.3 ± 1.4 | 3.6 ± 1.6 | 3.1 ± 1.6 | 2.6 ± 1.5 | 3.3 ± 1.6 | 3.3 ± 1.5 |

R: ROBUST, T: TRANSIENT, F: FRAIL
^1^formula: (Stride Length / 2) / (Number of Steps/min)
^2^the GAITRite system uses the term “Heel to Heel Base of Support”
^3^coefficient of variance; formula: (Standard Deviation / Mean) * 100

**Table S6: Supplementary Table 6c and Table 6d: Gait parameters at fast speed by functional ability, sex and age group 80y+**

| **Supplementary Table 6c: Gait parameters at fast speed by age 80y+, sex and Short Physical Performance Battery (SPPB) score** | | | | | | | | |
| --- | --- | --- | --- | --- | --- | --- | --- | --- |
| **Fast n=214** | **Male** | | | | **Female** | | | |
| **Functional ability** | **80y+ Total** | **80y+ Robust** | **80y+ Transient** | **80y+ Frail** | **80y+ Total** | **80y+ Robust** | **80y+ Transient** | **80y+ Frail** |
| **Gait Parameter** | n=79 | n=13 | n=35 | n=31 | n=135 | n=21 | n=56 | n=58 |
| 1. Velocity [cm/s] | 140.3 ± 28.8 | 163.1 ± 28.4 | 150.4 ± 19.9 | 119.3 ± 24.4 | 135.8 ± 25.6 | 159.2 ± 21.9 | 144.6 ± 18.7 | 118.9 ± 21.5 |
| 2. Stride length [cm] | 138.3 ± 20.8 | 152.5 ± 20.5 | 146.1 ± 14.2 | 123.5 ± 18.6 | 124.0 ± 17.0 | 136.8 ± 14.7 | 128.3 ± 14.0 | 115.2 ± 16.1 |
| 3. Walk ratio^1^ [cm/(steps/min)] | 0.57 ± 0.09 | 0.60 ± 0.11 | 0.60 ± 0.08 | 0.54 ± 0.08 | 0.48 ± 0.07 | 0.49 ± 0.07 | 0.48 ± 0.07 | 0.47 ± 0.07 |
| 4. Single support time [ms] | 376 ± 32 | 369 ± 39 | 373 ± 31 | 382 ± 29 | 350 ± 32 | 342 ± 31 | 343 ± 29 | 361 ± 32 |
| 5. Double support time [ms] | 250 ± 62 | 206 ± 41 | 235 ± 53 | 285 ± 61 | 227 ± 54 | 182 ± 29 | 209 ± 38 | 260 ± 55 |
| 6. Step width^2^ [cm] | 11.0 ± 3.5 | 10.0 ± 2.7 | 9.7 ± 2.6 | 12.9 ± 3.9 | 9.0 ± 3.1 | 8.8 ± 2.5 | 8.6 ± 2.9 | 9.4 ± 3.3 |
| 7. Step width^2^ CV^3^ [%] | 25.5 ± 17.1 | 26.7 ± 24.5 | 28.2 ± 17.3 | 22.0 ± 12.6 | 28.1 ± 16.8 | 26.1 ± 10.4 | 30.4 ± 18.8 | 26.7 ± 16.6 |
| 8. Stride length CV^3^ [%] | 4.7 ± 2.5 | 3.8 ± 2.9 | 4.6 ± 2.0 | 5.2 ± 2.8 | 4.1 ± 2.0 | 3.1 ± 2.0 | 3.9 ± 1.9 | 4.7 ± 1.8 |
|  | | | | | | | | |
| **Supplementary Table 6d: Gait parameters at fast speed by age 80y+, sex and LUCAS Functional Ability Index (LUCAS FAI)** | | | | | | | | |
| **Fast n=214** | **Male** | | | | **Female** | | | |
| **Functional ability** | **80y+ Total** | **80y+ Robust** | **80y+ Transient** | **80y+ Frail** | **80y+ Total** | **80y+ Robust** | **80y+ Transient** | **80y+ Frail** |
| **Gait Parameter** | n=79 | n=6 | n=49 | n=24 | n=135 | n=7 | n=67 | n=61 |
| 1. Velocity [cm/s] | 140.3 ± 28.8 | 168.1 ± 9.5 | 144 ± 28.2 | 125.7 ± 26.2 | 135.8 ± 25.6 | 148.4 ± 27.1 | 143.7 ± 24.2 | 125.7 ± 23.3 |
| 2. Stride length [cm] | 138.3 ± 20.8 | 159.2 ± 10.2 | 140.4 ± 18.5 | 128.8 ± 22.9 | 124.0 ± 17.0 | 128.6 ± 16.5 | 128.6 ± 16.7 | 118.3 ± 15.9 |
| 3. Walk ratio^1^ [cm/(steps/min)] | 0.57 ± 0.09 | 0.63 ± 0.09 | 0.58 ± 0.08 | 0.55 ± 0.10 | 0.48 ± 0.07 | 0.47 ± 0.06 | 0.48 ± 0.08 | 0.47 ± 0.06 |
| 4. Single support time [ms] | 376 ± 32 | 374 ± 39 | 374 ± 33 | 381 ± 29 | 350 ± 32 | 334 ± 33 | 348 ± 31 | 355 ± 32 |
| 5. Double support time [ms] | 250 ± 62 | 197 ± 23 | 244 ± 62 | 275 ± 59 | 227 ± 54 | 203 ± 46 | 212 ± 55 | 245 ± 49 |
| 6. Step width^2^ [cm] | 11.0 ± 3.5 | 8.7 ± 2.6 | 10.4 ± 2.6 | 12.8 ± 4.6 | 9.0 ± 3.1 | 9.2 ± 3.5 | 9.1 ± 3.2 | 8.9 ± 2.9 |
| 7. Step width^2^ CV^3^ [%] | 25.5 ± 17.1 | 22.8 ± 8.3 | 25.1 ± 16.0 | 27.0 ± 20.9 | 28.1 ± 16.8 | 25.0 ± 13.2 | 27.6 ± 14.0 | 29.0 ± 19.8 |
| 8. Stride length CV^3^ [%] | 4.7 ± 2.5 | 3.9 ± 1.8 | 4.2 ± 2.2 | 6.0 ± 2.9 | 4.1 ± 2.0 | 3.4 ± 2.1 | 4.0 ± 2.0 | 4.3 ± 1.9 |

R: ROBUST, T: TRANSIENT, F: FRAIL
^1^formula: (Stride Length / 2) / (Number of Steps/min)
^2^the GAITRite system uses the term “Heel to Heel Base of Support”
^3^coefficient of variance; formula: (Standard Deviation / Mean) * 100

**Table S7: Supplementary Table 7: Gender and age differences**

| **Gait parameters at preferred speed by age and Short Physical Performance Battery (SPPB) score** | | | | | | | |
| --- | --- | --- | --- | --- | --- | --- | --- |
| **Pref n=642** | **Male** | | | **Female** | | |  |
| **Functional ability** | **70-74y** | **75-79y** | **80+ y** | **70-74y** | **75-79y** | **80+ y** |  |
| **Gait Parameter** | n=51 | n=98 | n=84 | n=112 | n=147 | n=150 |  |
| 1. Velocity [cm/s] | 119.5 ± 29.3 | 116.4 ± 21.9 | 102.2 ± 23.9 | 121.8 ± 22.6 | 111.5 ± 21.5 | 99.7 ± 20.2 |  |
| 2. Stride length [cm] | 132.7 ± 24.9 | 129.9 ± 20.3 | 117.0 ± 21.2 | 126.3 ± 16.9 | 119.3 ± 16.2 | 108.4 ± 16.1 |  |
| 3. Walk ratio^1^ [cm/(steps/min)] | 0.62 ± 0.10 | 0.61 ± 0.10 | 0.56 ± 0.09 | 0.55 ± 0.06 | 0.54 ± 0.07 | 0.49 ± 0.07 |  |
| 4. Single support time [ms] | 408 ± 30 | 409 ± 32 | 411 ± 36 | 385 ±29 | 394 ± 35 | 393 ± 31 |  |
| 5. Double support time [ms] | 318 ± 91 | 310 ± 60 | 343 ± 74 | 281 ± 68 | 298 ± 64 | 318 ± 68 |  |
| 6. Step width^2^ [cm] | 11.0 ± 2.7 | 11.2 ± 3.1 | 11.9 ± 4.0 | 9.0 ± 3.4 | 9.0 ± 3.0 | 9.6 ± 3.2 |  |
| 7. Step width^2^ CV^3^ [%] | 20.1 ± 12.8 | 21.2 ± 12.9 | 22.4 ± 12.8 | 27.3 ± 17.4 | 27.7 ± 15.8 | 27.2 ± 15.0 |  |
| 8. Stride length CV^3^ [%] | 3.7 ± 1.7 | 3.9 ± 2.1 | 5.0 ± 2.8 | 3.3 ± 1.8 | 3.8 ± 1.8 | 4.7 ± 2.8 |  |

R: ROBUST, T: TRANSIENT, F: FRAIL
^1^formula: (Stride Length / 2) / (Number of Steps/min)
^2^the GAITRite system uses the term “Heel to Heel Base of Support”
^3^coefficient of variance; formula: (Standard Deviation / Mean) * 10
